# A CE/TA-stratified systematic review and evidence map of matcha and green tea bioactives in endocrine stress regulation: testosterone, cortisol, and the T:C ratio

**DOI:** 10.3389/fnut.2026.1925871

**Published:** 2026-09-08

**Authors:** Abdulmohsen Alanazi, Ryan Alanzi, Abdullah Abdulaziz Abbod Abdo

**Affiliations:** 1 Department of Basic Science, College of Science and Health Professions, King Saud bin Abdulaziz University for Health Sciences, Riyadh, Saudi Arabia; 2 King Abdullah International Medical Research Center, Riyadh, Saudi Arabia; 3 Ministry of National Guard-Health Affairs, Riyadh, Saudi Arabia; 4 College of Medicine, Shaqra University, Shaqra, Saudi Arabia; 5 Department of Applied Sciences, Faculty of Health and Life Sciences, Northumbria University, Newcastle upon Tyne, United Kingdom; 6 Department of Food Science and Technology, School of Food and Agriculture, Ibb University, Ibb, Yemen

**Keywords:** cortisol, EGCG, endocrine stress regulation, green tea, L-theanine, matcha, systematic review, testosterone-to-cortisol ratio

## Abstract

Matcha and green-tea bioactives are widely promoted for stress and hormonal health, yet the evidence is fragmented across chemically dissimilar preparations (whole-leaf matcha, brewed green tea, isolated L-theanine, and concentrated epigallocatechin-3-gallate, EGCG) that are routinely treated as interchangeable. A compositional index for this problem already exists: the caffeine-plus-EGCG-to-L-theanine-plus-arginine (CE/TA) molar ratio, developed by Unno and colleagues for matcha and Japanese green-tea preparations, together with an indicative screening threshold. This review adopts that index and applies it, for the first time, as a stratification variable across a formally assembled endocrine evidence base, in order to map what is and is not established regarding cortisol, testosterone, and the testosterone-to-cortisol (T:C) ratio. Following the Preferred Reporting Items for Systematic Reviews and Meta-Analyses (PRISMA) 2020 statement and its search extension (PRISMA-S), seven electronic sources were searched from inception to March 31, 2026, and findings were synthesized using the Synthesis Without Meta-analysis (SWiM) guideline because the studies were not sufficiently comparable for meta-analysis. Twenty-two studies were included (700 human participants; 673 endocrine-relevant), spanning randomized controlled trials, nonrandomized interventions, a cross-sectional study, animal experiments, and *in vitro* models. Studies were appraised with design-appropriate risk-of-bias tools and the Grading of Recommendations, Assessment, Development and Evaluation (GRADE) framework, and were stratified both by evidence tier and by CE/TA ratio. Lower-CE/TA preparations, particularly those around 2.0 or below, were the most consistently associated with stress-attenuating, cortisol-related responses, whereas higher-ratio and isolated-compound preparations were not. Catechin studies provided supportive but indirect evidence for testosterone preservation. Critically, no included matcha-specific human intervention measured testosterone and cortisol concurrently. GRADE certainty was low for cortisol-related and testosterone-related outcomes and very low for the integrated T:C endpoint. The contribution of this review is therefore 2-fold: the first tier-stratified application of the CE/TA index across an endocrine evidence base, and a formally documented map of the evidence gap, specifically the absence of chemically characterized trials with paired testosterone and cortisol measurement. Rather than a verdict that matcha is ineffective, these define a concrete, testable agenda for CE/TA-specified randomized trials with standardized sampling and paired endocrine endpoints.

**Systematic review registration:** https://inplasy.com/inplasy-2026-4-0072/, identifier INPLASY202640072.

## Introduction

Matcha and green-tea bioactives are among the most heavily promoted functional-food interventions for stress and hormonal health, yet the evidence underpinning these claims is unusually difficult to interpret. The relevant studies span chemically dissimilar preparations (whole-leaf matcha consumed as an aqueous suspension, filtered green-tea infusions, isolated L-theanine, and concentrated epigallocatechin-3-gallate (EGCG) extracts) that differ substantially in the very constituents thought to drive endocrine effects, yet are frequently pooled or treated as interchangeable. This compositional heterogeneity, rather than any single biological mechanism, may explain why the literature on green-tea bioactives and the stress-sensitive testosterone-to-cortisol (T:C) ratio appears inconsistent. The central premise of this review is that progress requires a framework that makes preparation composition explicit. We therefore organize the evidence using the caffeine-plus-EGCG-to-L-theanine-plus-arginine (CE/TA) molar ratio, a compositional index developed and applied to matcha and green-tea preparations by Unno and colleagues (13, 30–32), which pairs constituents associated with stress provocation against those associated with stress attenuation. The contribution of the present work is not the index itself, which is already established, but its systematic application as a prespecified stratification variable across a formally assembled endocrine evidence base, used to map, rather than overstate, what the current evidence can and cannot support.

Alterations in the T:C ratio have been used as indicators of anabolic-catabolic balance, with relevance to athletic recovery and physiological stress monitoring (1–3). Testosterone supports skeletal muscle protein synthesis, erythropoiesis, and positive nitrogen balance. In contrast, cortisol, the primary glucocorticoid released following activation of the hypothalamic-pituitary-adrenal (HPA) axis, promotes proteolysis, gluconeogenesis, and substrate mobilization during physiological stress (4, 5). Sustained HPA-axis activation can suppress hypothalamic-pituitary-gonadal (HPG) axis activity, thereby impairing gonadotropin signaling and testicular steroidogenesis (4, 5). Although the T:C ratio is not a standalone diagnostic marker, it remains useful as a longitudinal indicator of physiological stress, recovery status, and anabolic-catabolic homeostasis when interpreted alongside clinical, behavioral, and performance-related measures (2, 3, 5, 6). Recent endocrine literature has also highlighted its potential value as a practical, although non-diagnostic, tool for physiological stress monitoring and research across broader endocrine contexts (3). Detecting nutritional effects on this axis therefore requires careful control of intervention composition and endocrine biomarker measurement.

Interpretation of the T:C ratio requires explicit attention to how the index behaves and to what can distort it. The ratio is a unitless quotient in which the numerator and denominator must be expressed in the same units and derived from the same biological matrix; a higher value is conventionally read as relative anabolic predominance and a lower value as relative catabolic predominance, with within-person change over time being more informative than any single cross-sectional value, since no validated reference range or clinical decision threshold exists (2, 3, 5, 6). Several factors can move the ratio independently of any intervention. Cortisol follows a pronounced diurnal rhythm with a steep post-awakening rise and evening nadir, and testosterone shows a parallel but smaller morning peak, so unstandardized sampling time can change the ratio severalfold (36, 38, 39). Serum and salivary measurements are not interchangeable, because saliva approximates the free fraction while serum assays typically report total hormone; sex hormone-binding globulin, albumin, and corticosteroid-binding globulin therefore alter the relationship between total and bioavailable concentrations, and immunoassay and mass-spectrometry platforms are not numerically equivalent (36–39). Acute and anticipatory stress, exercise intensity and time since the last training session, sleep duration, energy availability, adiposity, age, pubertal stage, menstrual-cycle phase or hormonal contraceptive use, and acute caffeine intake each perturb one or both hormones and hence the ratio (1, 2, 5, 6, 70, 71). Because the ratio is a quotient, imprecision in either hormone propagates into it, so assay variability is amplified rather than averaged out. These constraints are the reason the present review treats the T:C ratio as a prespecified, matrix-matched, and time-standardized endpoint, and does not derive it *post-hoc* by combining hormone values measured in different studies, matrices, or sampling frameworks.

Nutritional strategies that mitigate excessive stress responses while maintaining anabolic signaling are increasingly important in functional-food and sports-nutrition research. Matcha, a finely milled whole-leaf preparation of shade-grown *Camellia sinensis*, is notable because it is consumed as an aqueous suspension rather than a filtered infusion. This method of consumption may enhance exposure to catechins, including epigallocatechin-3-gallate (EGCG), as well as L-theanine, caffeine, and fiber-associated phenolics (7–10). Human studies have investigated matcha in relation to mood, cognition, fatigue, and stress-related outcomes; however, its endocrine effects, particularly regarding concurrent cortisol and testosterone regulation, have not been comprehensively synthesized (11–13).

The rationale for investigating matcha in endocrine stress regulation is supported by the activities of L-theanine and EGCG. L-theanine crosses the blood-brain barrier and modulates glutamatergic and GABAergic neurotransmission, with human studies reporting reductions in subjective or physiological stress responses under selected experimental conditions (14–17). However, systemic cortisol outcomes vary across trials, likely due to differences in stress paradigms, sampling times, biological matrices, and intervention compositions (18–21). EGCG has also been studied for endocrine-relevant effects within a broader context of antioxidant, anti-inflammatory, and immunomodulatory activities pertinent to stress physiology (22). Mechanistic studies indicate that EGCG may inhibit 11β-hydroxysteroid dehydrogenase type 1 (11β-HSD1), an enzyme responsible for regenerating active cortisol from cortisone in metabolically active tissues (23, 24). Experimental models further suggest that catechins may influence Leydig-cell steroidogenesis, although these effects are dose-dependent. Direct extrapolation to dietary matcha exposure is not justified because experimental models often employ pharmacological concentrations (25, 26). Catechins may also affect androgen availability through modulation of testosterone metabolism and glucuronidation, although this evidence remains indirect for dietary matcha (27). Translation from isolated or high-dose experimental systems to human dietary exposure is further constrained by the low systemic bioavailability of native EGCG and the potential influence of gut microbiome-mediated catechin biotransformation (10, 28, 29).

These considerations highlight the importance of evaluating matcha as a complex formulation rather than merely as a source of isolated bioactives. The physiological effects of green-tea preparations may depend in part on the balance between components that may counteract stress-attenuating effects, such as caffeine and EGCG, and compounds associated with stress attenuation, such as L-theanine and arginine. The caffeine-plus-EGCG-to-L-theanine-plus-arginine molar ratio (CE/TA ratio), which was proposed and developed by Unno and colleagues, may serve as a useful compositional index for interpreting heterogeneity across studies (13, 30–32). In matcha-focused animal and human studies, lower CE/TA ratios, typically around 2.0 or below, have been associated with stress attenuation (13, 30). Broader analyses of Japanese green-tea preparations have proposed a screening threshold below 3.0 (31, 32). These values should be regarded as exploratory, formulation-dependent thresholds rather than validated clinical cutoffs. Given the substantial compositional variability among commercial matcha products, incomplete reporting and limited stratification by CE/TA ratio may contribute to inconsistent findings in the functional-nutrition literature (10, 30–34).

Direct clinical evidence linking standardized matcha consumption to integrated changes in the human T:C ratio is currently limited. The most directly relevant intervention to date reported that daily matcha intake during resistance training was associated with a lower change in salivary cortisol secretion rate and moderated fatigue; however, testosterone was not measured, precluding calculation of the T:C ratio (11). More broadly, studies of green-tea bioactives and endocrine outcomes differ in intervention composition, biological matrices, assay methodologies, sampling times, and control of circadian variation (11, 18–21, 35–40). These methodological differences complicate cross-study comparisons and may contribute to inconsistent endocrine findings. The primary evidence gap is not merely the limited number of matcha trials, but the lack of chemically characterized, CE/TA-specified interventions that concurrently measure testosterone and cortisol using standardized sampling protocols. Thus, whether matcha-specific interventions modulate the human T:C ratio remains unconfirmed.

The CE/TA index itself has been described and applied to individual green-tea and matcha preparations by Unno and colleagues (13, 30–32). To our knowledge, however, no previous systematic review has used it as a stratification variable while integrating matcha formulation chemistry, cortisol-related outcomes, testosterone-related outcomes, and evidence relating to the T:C ratio within a unified endocrine stress-regulation framework. This systematic review synthesizes mechanistic and clinical evidence on matcha and green-tea bioactives in relation to endocrine stress regulation. Specifically, it aims to: (1) evaluate the effects of matcha, EGCG, and L-theanine on cortisol and HPA-axis-related outcomes; (2) assess evidence for testosterone-related effects of green-tea bioactives across relevant populations; and (3) determine whether direct evidence exists for modulation of the T:C ratio. By integrating formulation characteristics through the CE/TA framework and identifying biomarker, chronobiological, and methodological limitations, this review delineates the current evidence gap and provides a translational framework for future randomized controlled trials.

## Chemical characterization of matcha and its bioactive constituents

Matcha is a chemically complex whole-leaf preparation containing polyphenols, amino acids, methylxanthines, pigments, and dietary fiber. Catechins are major polyphenolic constituents, with EGCG among the major catechins (7, 8, 10, 33, 41, 42). However, the composition of matcha is not uniform. Cultivar, shading conditions, harvest timing, leaf maturity, processing, storage, grade, preparation method, and analytical approach can influence the abundance and relative proportions of its bioactive constituents (7, 8, 10, 33, 34, 41–43).

**Figure 1:**
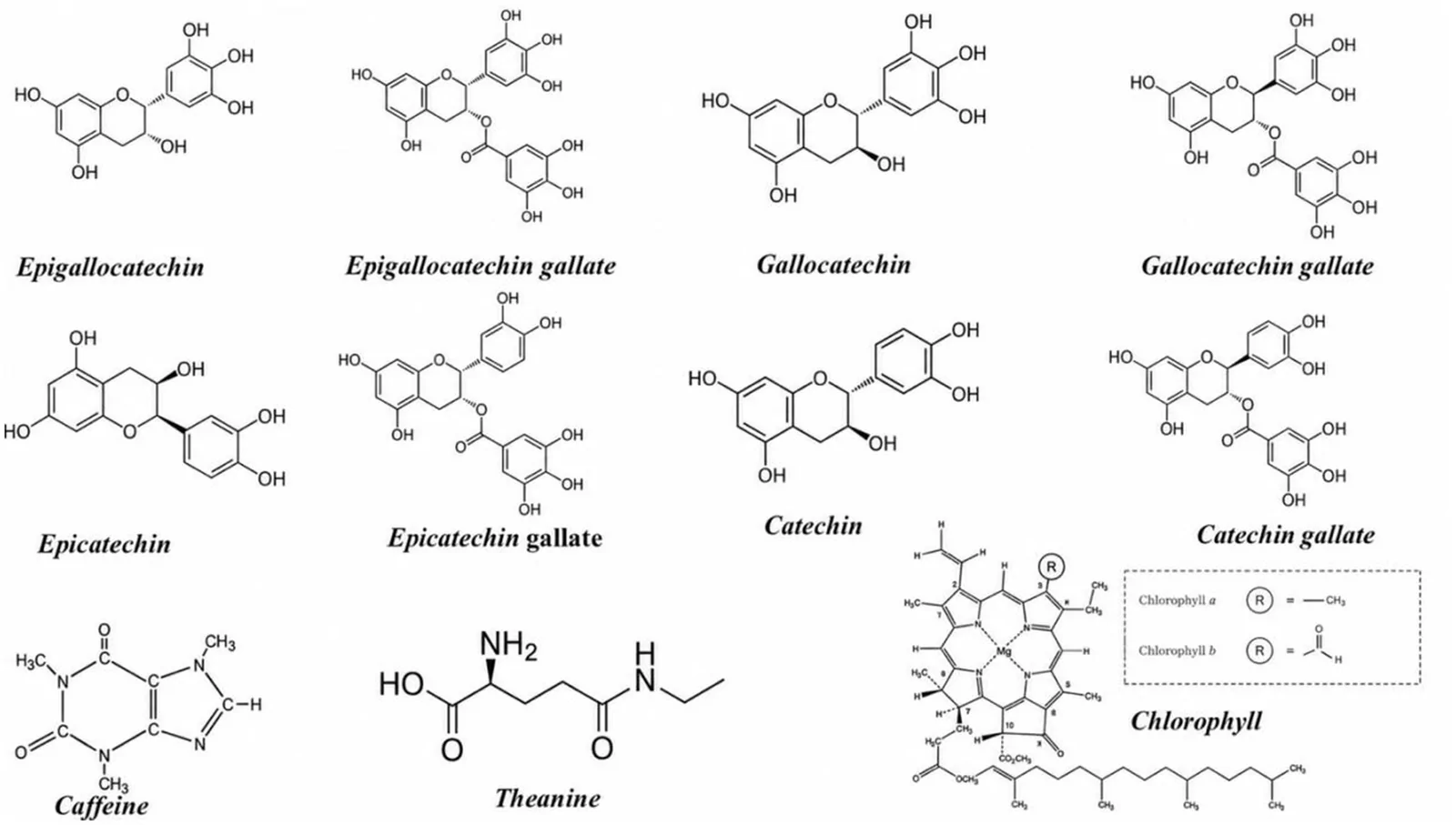
Major bioactive constituents of matcha (*Camellia sinensis*). Chemical structures represent the primary molecular classes discussed in this review, including the eight major green tea catechins (epigallocatechin, epigallocatechin gallate, gallocatechin, gallocatechin gallate, epicatechin, epicatechin gallate, catechin, and catechin gallate), the methylxanthine alkaloid caffeine, the non-proteinogenic amino acid L-theanine, and the primary leaf pigment chlorophyll. For the porphyrin macrocycle of chlorophyll, the functional R group denotes the structural distinction between chlorophyll a (-CH3) and chlorophyll b (-CHO).

Product-level analyses provide a more informative basis for estimating potential dietary exposure than reliance on a single assumed serving composition. Meyer et al. reported EGCG concentrations ranging from 36.70 to 69.73 mg/g in commercially available matcha products (33), corresponding to approximately 73 to 139 mg per 2 g serving. In the same analysis, caffeine concentrations ranged from 17.74 to 35.01 mg/g, equivalent to approximately 35 to 70 mg per 2 g serving (33). Other studies have reported lower sample-specific caffeine values, further demonstrating the influence of product characteristics and analytical context (42, 44). L-theanine content also varies among matcha products and grades (7, 30, 34, 42). Selected bioactive constituents of matcha are presented in Figure 1.

A key distinction between matcha and steeped green tea is their mode of consumption. Matcha is consumed as a whole-leaf aqueous suspension rather than as a filtered infusion, permitting exposure to both soluble constituents and less readily extractable matrix components. These include dietary fiber, chlorophyll, and other matrix-associated phytochemicals (7, 9, 10). In one analysis of dry matcha powder, total dietary fiber accounted for 56.1 g/100 g, of which 94.1% was insoluble fiber (44). Although these values are sample-specific, they point to the importance of evaluating matcha as a whole-food matrix rather than solely as a source of isolated catechins. Matrix-associated compounds may influence gastrointestinal bioaccessibility and microbiome-mediated biotransformation, with potential implications for systemic exposure (10, 28, 29, 42).

The physiological effects of matcha cannot be inferred solely from absolute catechin concentrations. Stress-related outcomes may depend partly on the relative balance between the denominator components, L-theanine and arginine, and the numerator components, caffeine and EGCG. The molar ratio of caffeine and EGCG to L-theanine and arginine, referred to as the CE/TA ratio, has been proposed as an exploratory compositional index for interpreting heterogeneity across matcha and green-tea studies (13, 30–32). Matcha-focused animal and human studies have reported stress attenuation at CE/TA ratios of approximately 2.0 or lower (13, 30), whereas broader analyses of Japanese green-tea preparations have suggested a screening threshold below 3.0 (31, 32). These values remain formulation-dependent and should be interpreted as hypothesis-generating rather than clinically validated cutoffs.

Caution is required when extrapolating findings from experimental models to human dietary interventions. Preclinical studies often use purified EGCG or concentrated green-tea polyphenol extracts at pharmacological or supraphysiological doses, whereas human studies generally evaluate whole-food preparations or isolated bioactives within human intervention protocols (25, 26, 28, 29, 45). Animal and *in vitro* findings should therefore be interpreted as mechanistic support rather than as direct predictors of clinical efficacy at dietary matcha doses.

Within this review, intervention-specific reporting of catechins, amino acids, methylxanthines, grade, preparation conditions, and analytical methods is essential for interpreting endocrine-related outcomes and improving reproducibility in future clinical trials.

## Methods

### Protocol and registration

This systematic review was conducted and reported in accordance with the Preferred Reporting Items for Systematic Reviews and Meta-Analyses (PRISMA) 2020 statement (46). Reporting of the literature search was additionally aligned with the PRISMA-S extension (47). The review was retrospectively registered with the International Platform of Registered Systematic Review and Meta-analysis Protocols (INPLASY; registration number: INPLASY202640072). Registration occurred after the initial literature search and preliminary screening had begun; however, the eligibility criteria, extraction framework, and synthesis approach were finalized before formal data extraction and evidence synthesis. Protocol deviations and post-registration analytical decisions are disclosed transparently below.

### Eligibility criteria

Study selection was guided by an adapted PICOS framework to accommodate the heterogeneous evidence base. Eligible human populations included participants regardless of health status. Human studies reporting serum or salivary testosterone, cortisol, or the T:C ratio were considered the most directly relevant endocrine evidence. Human studies reporting stress-related, autonomic, or metabolically relevant outcomes were retained as supportive evidence when these outcomes informed the interpretation of matcha or green-tea bioactives. Relevant animal models and *in vitro* systems reporting hormonal regulation, stress physiology, cortisol metabolism, steroidogenesis, or mechanistically relevant endocrine pathways were included as mechanistic evidence. Animal and *in vitro* studies were interpreted as supportive evidence of biological plausibility and were not considered direct evidence of clinical efficacy. The complete eligibility framework, including the operational inclusion and exclusion rule applied at every PICOS element, is set out in structured form in Supplementary Table S8.

Eligible interventions comprised whole-matcha preparations, matcha-containing products, standardized *Camellia sinensis* preparations, brewed green tea, green-tea extracts, green-tea polyphenols, and isolated green-tea bioactives, including EGCG, L-theanine, and caffeine. This broader intervention scope was retained because direct whole-matcha endocrine trials were limited, while related green-tea bioactive studies provided mechanistically relevant evidence for interpreting cortisol, testosterone, and biology of the T:C ratio. Eligible comparators included placebo, water, no intervention, or alternative beverages. Primary outcomes were serum or salivary testosterone, cortisol, and the T:C ratio. Secondary and mechanistic outcomes included markers of HPA axis and hypothalamic-pituitary-gonadal (HPG) axis biology. Studies cited solely to contextualize matcha chemistry, product variability, bioaccessibility, or analytical methods were not included in the formal endocrine evidence count unless they independently met the eligibility criteria.

### Evidence tier assignment and allocation to synthesis

Each included study was assigned to exactly one of four mutually exclusive evidence tiers, applied by two reviewers independently at the point of data extraction and reconciled by consensus. Tier 1, direct clinical evidence, comprised human interventional studies in which the administered product was whole matcha or a matcha-containing preparation and in which testosterone, cortisol, or the T:C ratio was a measured endpoint. Tier 2, supportive human evidence, comprised human studies that measured testosterone or cortisol but administered a non-matcha green-tea preparation or an isolated bioactive, together with human studies of any tea preparation that reported only stress-related proxy endpoints such as salivary α-amylase, heart-rate variability, or electroencephalographic measures. Tier 3, animal evidence, comprised controlled *in vivo* animal experiments reporting endocrine, stress-physiological, or reproductive endpoints. Tier 4, *in vitro* evidence, comprised cell-free, microsomal, or cell-culture systems addressing steroidogenic or prereceptor glucocorticoid pathways. Where a single publication reported both a human and an animal component, the study was assigned to the higher tier for the purpose of the human evidence count and its animal component was appraised separately with the animal-study tool. Tier assignment governed both the risk-of-bias instrument applied and the interpretive weight given in synthesis: only Tier 1 and Tier 2 contributed to the human participant totals and to the GRADE assessment of human outcomes, whereas Tier 3 and Tier 4 informed biological plausibility and the GRADE indirectness domain and were never used to support inferences about clinical efficacy. Allocation of every included study to its tier, and to each of the three review aims, is reported in Supplementary Table S9.

### Information sources and search strategy

Seven electronic sources were searched from inception to March 31, 2026: PubMed/MEDLINE, Scopus, Embase, Cochrane CENTRAL, Dimensions, ScienceDirect, and SpringerLink. PubMed/MEDLINE, Scopus, Embase, Cochrane CENTRAL, and Dimensions were used as bibliographic or indexing sources, while ScienceDirect and SpringerLink were searched as publisher platforms to broaden retrieval. Controlled vocabulary was applied where supported and supplemented by free-text terms related to matcha, green tea, Camellia sinensis, EGCG, catechins, L-theanine, caffeine, testosterone, cortisol, stress, and endocrine regulation. Searches were restricted to English-language publications. Complete source-specific search strategies are provided in Supplementary Table S7, which reports, for each source, the host platform or interface, the exact date on which the search was executed, the full executed search string as run, every filter and limit applied, and the number of records retrieved. No date restriction, publication-type filter, subject-area filter, or document-type limit was applied at any source; the single restriction applied uniformly was to English-language publications.

The source selection differed from the registered protocol, which listed Web of Science and Google Scholar. Web of Science was not accessible during the search period, and Google Scholar was not searched systematically, consistent with evidence that it is less suitable as a primary systematic-review search tool than dedicated bibliographic databases (48). Cochrane CENTRAL, Embase, and Dimensions were added to broaden retrieval. Two considerations bear on whether this source set was adequate. First, the indexing scope of the seven sources searched overlaps substantially with that of Web of Science: Scopus and Dimensions together index the great majority of journals covered by the Web of Science Core Collection in nutrition, endocrinology, and food science, and Dimensions additionally indexes full text rather than metadata alone. Second, the retrieval itself provides empirical evidence of near-saturation, in that hand-searching the reference lists of every included study and of all matcha-focused reviews identified during screening yielded only four additional records, against 2,796 retrieved by database searching. A yield of this magnitude from citation chasing is consistent with a search that had already approached saturation for the eligible literature, although it cannot exclude the possibility that individual records were missed. Trial registries such as ClinicalTrials.gov and the World Health Organization International Clinical Trials Registry Platform, and gray-literature sources such as theses and conference proceedings, were not searched, because eligibility was restricted to peer-reviewed publications reporting extractable primary data; the resulting potential for reporting bias is acknowledged as a limitation. Reference lists of included studies and relevant reviews were screened manually to identify additional eligible records. No formal gray-literature search was conducted. This limitation was considered when interpreting the comprehensiveness of the evidence base and the potential for publication bias.

### Study selection

After deduplication in Rayyan (49), two reviewers (AA and RA) independently screened titles and abstracts. Full-text articles were subsequently assessed independently against the eligibility criteria. Disagreements were resolved through structured consensus. Inter-rater agreement was substantial at the title and abstract screening stage (Cohen's κ = 0.82) and near-perfect at full-text assessment (κ = 0.91). Final eligibility screening identified 22 included studies, superseding the preliminary INPLASY count of 16 after updated searching and full-text assessment. One study enrolling male adolescents was retained because it reported relevant endocrine outcomes, although it was interpreted cautiously because pubertal stage may influence hormonal measures.

### Data extraction

Data extraction was performed independently by two reviewers using a standardized extraction form. Extracted variables included study design, population or model characteristics, intervention composition, dose, duration, comparator, biological matrix, assay methodology, sampling time, chronobiological considerations, and quantitative endocrine outcomes. Discrepancies were resolved through consensus.

## CE/TA stratification framework

Where sufficient compositional data were available, interventions were categorized according to the molar ratio of caffeine and EGCG to L-theanine and arginine, referred to as the CE/TA ratio and defined in Equation 1:

CE/TA ratio=(moles of caffeine + moles of EGCG)/(moles of L-theanine + moles of arginine) (1)

This framework was introduced after registration as an exploratory analytical approach, based on prior mechanistic and intervention literature indicating that lower CE/TA ratios may be more consistently associated with stress attenuation (13, 30–32). Matcha-focused interventions were categorized primarily according to whether the CE/TA ratio was approximately 2.0 or lower or above 2.0. The broader threshold below 3.0 proposed for Japanese green-tea preparations was considered contextually rather than as the primary classification threshold (32).

Study-level CE/TA values were classified as exact when all four constituent values were reported directly or when the complete ratio was reported by the original study, and as fully estimated when a complete ratio could be derived from study-specific compositional data. Values were classified as partially estimated when one or more constituents were unavailable and as non-calculable when the available information was insufficient. Standard compositional averages were not used to impute missing values. Only exact or fully estimated ratios were used for categorical stratification. Because CE/TA stratification was not prespecified in the registered protocol, the findings were interpreted as exploratory and hypothesis-generating rather than confirmatory. Study-level calculations, classifications, and assumptions are reported in Supplementary Table S6.

## Risk-of-bias assessment

Risk-of-bias and critical-appraisal methods were selected according to study design, as summarized in Supplementary Table S1. Randomized controlled trials were evaluated using the Cochrane Risk of Bias 2 tool (50) for the primary review-relevant outcome reported in each trial rather than for the study as a whole. Non-randomized intervention studies were assessed using ROBINS-I (51), cross-sectional studies using an adapted Newcastle-Ottawa Scale (52), and animal studies using the complete 10-domain SYRCLE tool (53). Mechanistic *in vitro* studies were appraised narratively for methodological validity, including the physiological relevance of dosing, and were incorporated into the assessment of indirectness rather than assigned a formal clinical risk-of-bias category. Risk-of-bias assessments were completed independently by two reviewers, and disagreements were resolved through consensus. Risk-of-bias judgments are reported in Supplementary Tables S2–S5.

## Data synthesis

Formal meta-analysis was not performed because the included studies were not sufficiently exchangeable to support a clinically interpretable pooled estimate. The evidence base differed substantially across intervention type, population, study design, biological matrix, hormonal assay, sampling time, stress context, and outcome definition. Pooling isolated L-theanine, brewed green tea, whole matcha, catechin co-ingestion, animal models, and mechanistic *in vitro* systems would have produced a summary estimate with limited biological and clinical meaning. A structured narrative synthesis was therefore conducted in accordance with the Synthesis Without Meta-analysis (SWiM) reporting guideline (54). Evidence was grouped by outcome domain: cortisol and HPA-axis-related outcomes, testosterone and HPG-axis-related outcomes, and the integrated T:C endpoint. Within each domain, studies were interpreted according to intervention class and evidence tier. Greater interpretive weight was given to human randomized controlled trials and interventions most directly relevant to whole matcha, while green-tea extracts, isolated bioactives, observational studies, animal models, and *in vitro* systems were considered indirect or mechanistic evidence.

Where individual studies did not report the T:C ratio, *post-hoc* calculation was planned only when paired testosterone and cortisol data were available from the same participants or experimental units and measured within a compatible sampling framework. Values were not combined across separate studies, cohorts, biological matrices, or time points. Because most included studies did not measure testosterone and cortisol concurrently, robust pooled *post-hoc* analysis of the T:C ratio was not feasible.

## Certainty of evidence (GRADE)

The certainty of evidence was assessed using the Grading of Recommendations, Assessment, Development and Evaluation (GRADE) framework (55). Certainty was evaluated separately by outcome and evidence tier, considering risk of bias, inconsistency, indirectness, imprecision, and publication bias. Animal and *in vitro* evidence informed biological plausibility and indirectness but did not increase the certainty of conclusions regarding human clinical efficacy. The GRADE summary is reported in Table 1.

**Table 1:** GRADE summary of evidence certainty for primary outcomes.

Outcome	Studies (*n*)	Participants	Risk of bias	Inconsistency	Indirectness	Imprecision	Certainty (GRADE)	Rationale and key downgrading factors
Cortisol or cortisol-related stress outcomes in human studies	7 human intervention studies/components	214	Serious: one study at serious risk of bias (ROBINS-I) and three randomized trials with some concerns	Not serious, generally consistent direction	Serious indirectness due to reliance on L-theanine/green tea studies rather than direct whole-matcha T:C trials.	Serious, small samples across studies	LOW	Downgraded for indirectness and imprecision due to heterogeneous interventions/outcomes and limited concurrent testosterone assessment, despite evidence of reduced stress-related biomarkers. Seven human intervention studies assessed cortisol or stress-related outcomes (Dini and Pourrazi, Almudhi and Gabr, Shigeta, Evans, White, Moulin, and Lim; total *N* = 214). Kimura et al. (16) and Yoto et al. (17) are not included in this cortisol GRADE participant count because cortisol was not a primary measured endpoint in those trials.
Testosterone-related outcomes in human studies	2 intervention studies and 1 cross-sectional controlled study	389	Serious, includes one uncontrolled pre-post study and one observational study	Serious, heterogeneous populations and intervention types	Serious, evidence mainly from green tea or catechin-based exposure rather than whole matcha	Serious, limited number of studies and variable designs	LOW	Downgraded for risk of bias, inconsistency, indirectness, and imprecision; evidence suggests possible testosterone benefits from green tea, but causal inference remains limited.
Testosterone-to-cortisol ratio as an integrated endpoint	Few studies with paired hormone data	Not pooled	Serious	Serious	Very serious, most studies measured only one hormonal component	Very serious, few paired testosterone and cortisol data	VERY LOW	Downgraded for indirectness, imprecision, and heterogeneity, as most studies did not assess testosterone and cortisol concurrently; evidence for T:C modulation remains indirect.
Cortisol or stress-related outcomes in animal studies	4 animal studies	Not pooled	Low to moderate, SYRCLE applied	Serious, different species and stress models	Very serious, animal models and non-human dosing	Serious, small animal studies	VERY LOW	Animal studies support stress- and cortisol-lowering effects of matcha-related compounds, but direct extrapolation to human T:C modulation remains limited.
Metabolic, reproductive, and mechanistic outcomes in animal studies	4 animal studies	Not pooled	Low to moderate, SYRCLE applied	Serious, different models and endpoints	Very serious, animal models and indirect endpoints	Serious, small number of studies	VERY LOW	Animal and mechanistic studies support matcha-related metabolic, stress, and gene-expression effects, but direct evidence for human testosterone, cortisol, or T:C modulation is lacking.

The participant column reports the number enrolled in each contributing study, for consistency with Table 3. Where an outcome was assessed in only a subset of those participants, the subset is stated in Table 3 and in Supplementary Table S10; the principal instances are Shigeta et al., in which salivary cortisol was measured in trial 2 only (n = 19 of 36), and Onuora et al., in which 40 of the 100 screened participants received the supplementation. Counting only the participants contributing directly to each outcome would give 197 for the cortisol outcome and 329 for the testosterone outcome; neither substitution alters any certainty rating, because both outcomes were already downgraded for imprecision. GRADE certainty ratings: High = further research is very unlikely to change confidence in the effect estimate; Moderate = further research is likely to have an important impact on confidence; Low = further research is very likely to have an important impact; Very Low = very uncertain about the estimate. Downgrading criteria were applied according to standard GRADE guidelines. Cortisol-related and testosterone-related outcomes were graded separately because most studies did not measure both hormones within the same intervention. T:C evidence was downgraded for indirectness where only one hormonal component was measured. RoB, risk of bias; T:C, testosterone-to-cortisol ratio; CE/TA, molar ratio of caffeine and EGCG to L-theanine and arginine.

## Differences between the protocol and the review

Because registration was retrospective, several differences between the registered INPLASY protocol (INPLASY202640072) and the completed review are disclosed here in the interest of transparency. First, the registered protocol specified five bibliographic databases (PubMed, Scopus, ScienceDirect, SpringerLink, and Web of Science Core Collection) with Google Scholar searched supplementarily; the completed review instead searched seven electronic sources (PubMed, Scopus, ScienceDirect, Cochrane CENTRAL, SpringerLink, EMBASE, and Dimensions). Web of Science was not accessible during the search period and was therefore not searched, and Google Scholar was not searched systematically; Cochrane CENTRAL, EMBASE, and Dimensions were added to broaden retrieval. Second, the registered protocol described a preliminary tabular summary of 16 studies; following completion of screening and full-text assessment of all records identified by the final search, the included evidence set was finalized at 22 studies. No new searches were run after the registered search dates, and all included studies were identified within the original search and screening process; the change reflects completion of the screening workflow rather than the addition of records from a later search. Third, the registered protocol referred to the index as the testosterone-to-cortisol (T/C) ratio; the manuscript uses the equivalent notation T:C ratio throughout for consistency with the registered title and the predominant convention in the endocrine literature. Fourth, risk of bias for the *in vitro* studies was appraised using a narrative critical-appraisal approach rather than the ToxRTool named in the protocol, and the single non-randomized pre-post study by Almudhi and Gabr, initially considered alongside controlled designs, was appraised using ROBINS-I appropriate to its uncontrolled design. Fifth, the CE/TA molar ratio, described in the protocol as a formulation parameter, is presented in the manuscript as an exploratory, post-registration, hypothesis-generating stratification rather than a validated threshold. Sixth, the manuscript title was revised after registration to reflect more precisely the methodological contribution (the CE/TA stratification framework) and the evidence-mapping scope of the review; the registered title had described the index as being modulated, whereas the completed review frames matcha and green-tea bioactives as biologically plausible but not clinically confirmed in this role. This revision did not change the prespecified review question, the eligibility criteria, or the direction of the conclusions. Seventh, the two *in vitro* reports by Hintzpeter et al. (23) and Szelényi et al. (24) are counted, appraised, and tabulated as two separate included studies, consistent with their status as independent publications; the total number of included studies is accordingly reported as 22, with no change to the human participant totals, the risk-of-bias judgments, or any outcome-level finding. None of these changes altered the prespecified review question, eligibility criteria, or the conclusion that direct clinical evidence for modulation of the T:C ratio remains insufficient.

**Table 2:** Database search strings and record counts.

Database/Source	Search String (abbreviated)	Records (*n*)
PubMed (MEDLINE)	(“matcha” OR “*Camellia sinensis*” OR “green tea”) AND (EGCG OR epigallocatechin OR theanine OR catechin) AND (testosterone OR cortisol OR “hydroxysteroid dehydrogenase”)	18
Scopus	TITLE-ABS-KEY (“matcha” OR “EGCG” OR “L-theanine”) AND (testosterone OR cortisol OR “T:C ratio”))	61
ScienceDirect	“matcha” AND (“testosterone” OR “cortisol”)	7
Cochrane CENTRAL	(“green tea” OR “matcha”) AND “cortisol”	1
SpringerLink	(“*Camellia sinensis*” OR “green tea”) AND (testosterone OR cortisol)	18
EMBASE	(matcha OR EGCG OR theanine) AND (testosterone OR cortisol)	555
Dimensions	(matcha OR “green tea” OR “*Camellia sinensis”)* AND (EGCG OR epigallocatechin OR theanine OR catechin) AND (testosterone OR cortisol OR “hydroxysteroid dehydrogenase”)	2,136
Hand-searching	Manual review of reference lists of all included studies and relevant matcha-focused reviews	4
Web of Science	Not accessible during search period	N/A
Total		2,800

All searches were conducted between January and March 2026, with the final search completed on March 31, 2026. No date restrictions were applied, and searches were limited to English-language publications. Search strings are presented in abbreviated form for readability; full database-specific search strategies are provided in Supplementary Table S7. EMBASE and Dimensions produced broad retrievals, with 555 and 2,136 records, respectively. These broad searches were retained to increase bibliographic sensitivity and were managed through duplicate removal followed by two-stage title/abstract and full-text screening.

**Table 3:** Characteristics and key findings of the 22 included studies.

No.	Reference	Design	*N*	Population/model	Duration	Intervention	Key findings relevant to T:C	RoB
1	Kimura et al. (16)	RCT crossover	12	Healthy adult males	Single dose	L-theanine 200 mg	Physiological stress responses were attenuated. Testosterone was not measured; T:C ratio could not be calculated.	Some concerns
2	Yoto et al. (17)	RCT crossover	14	Healthy adults, mixed sex	Acute	L-theanine 200 mg vs. placebo	Blood pressure and physiological stress responses were attenuated. T:C ratio could not be calculated.	Low
3	Scholey et al. (56)	RCT crossover	27	Healthy adults	Acute	EGCG	Acute mood- or stress-related effects were reported. Cortisol and testosterone were not measured; T:C ratio could not be calculated.	Low
4	Suzuki et al. (63)	RCT crossover	9	Well-trained male cyclists	Acute	Green tea catechins plus carbohydrate, 22 mg/kg catechins	Testosterone was maintained compared with the significant decline observed in carbohydrate-only controls (p < 0.001). Cortisol was not significantly altered; T:C interpretation remains indirect.	Low
5	Dini and Pourrazi (40)	RCT parallel	20	Young women with anxiety (ages 18–22 years)	1 week	Green-tea leaf powder, 1,000 mg/day vs. control	Serum cortisol did not differ significantly between groups (p = 0.96). Serotonin increased and rate-pressure product decreased. Testosterone was not measured; T:C ratio could not be calculated.	Low
6	Shigeta et al. (11)	RCT parallel	36 across two trials; 19 in the cortisol trial	Healthy untrained males	8 to 12 weeks	Matcha beverage, 1.5 g twice daily	The change in salivary cortisol secretion rate was significantly lower in the matcha group (p < 0.05), and fatigue was moderated. Testosterone was not measured; T:C ratio could not be calculated.	Low
7	Sae-tan et al. (60)	Animal experiment	36	Male C57BL/6 mice	16 weeks	Dietary EGCG, 0.32%	Fat-oxidation gene expression increased and metabolic markers improved. No direct testosterone, cortisol, or T:C outcome was assessed.	Low
8	Chen et al. (45)	Animal experiment	40	Male Wistar rats	3 weeks	Green tea polyphenols or EGCG, 0.1 g/day	EGCG and green tea polyphenols attenuated stress-induced plasma cortisol elevation and improved behavioral impairment. Testosterone, StAR protein, and Leydig cell ROS were not assessed.	Low
9	Almudhi and Gabr (35)	Pre-post intervention study	60	Male adolescents	6 weeks	Decaffeinated green tea, 6 cups/day	Cortisol, DHEA, ACTH, and corticosterone were reduced (p < 0.05). Testosterone was not measured; T:C ratio could not be calculated.	Serious, ROBINS-I
10	Onuora et al. (64)	Nonrandomized pre-post intervention	100 screened; 40 supplemented	Obese adult males	12 weeks	Green tea, 2 bags/day	Testosterone increased (p < 0.05), estradiol and prolactin decreased, oxidative stress improved, and LH/FSH partly normalized. Cortisol was not measured; T:C ratio could not be calculated.	High
11	Wan et al. (65)	Cross-sectional controlled study	280	Middle-aged and older adult males	Long-term habitual intake	Green tea	Long-term green tea intake was associated with higher testosterone (574.66 vs. 495.89 ng/dL; p = 0.001) and lower inflammation. Cortisol was not measured; T:C ratio could not be calculated.	Moderate
12	Unno et al. (13)	Human RCT and animal experiment	36 pharmacy students; 40 mice	Pharmacy students and stressed male mice	15 days in humans; 7 days in mice	Matcha cookies, 4.5 g/day, CE/TA 1.79 vs. 10.64; matcha components in mouse diet	Salivary alpha-amylase was reduced in the low CE/TA matcha-cookie group. Adrenal hypertrophy was suppressed in stressed mice when CE/TA was close to 2. Testosterone and cortisol were not both measured; T:C ratio could not be calculated.	Low (human component)
13	Monobe et al. (59)	Animal experiment	8 to 10 mice/group	Male ddY mice exposed to social stress	2 weeks	Matcha, low-caffeine matcha, or sencha extract by oral administration	Continued matcha ingestion suggested reduced anxiety-like behavior after social stress. Cortisol and testosterone were not measured; T:C ratio could not be calculated.	Moderate
14	Mohamed et al. (58)	Animal experiment	18	Growing male New Zealand white rabbits	9 weeks	Matcha tea in drinking water, 0.0125 g/L	Plasma cortisol and insulin were reduced in the matcha group. Growth performance and metabolic gene expression improved. Testosterone was not measured; T:C ratio could not be calculated.	Moderate
15	Soliman et al. (66)	Animal experiment	18	Growing male New Zealand white rabbits	10 weeks	Matcha tea in drinking water, 0.037 g/3 L	Matcha modulated testicular expression of puberty- and fertility-related genes, including *DMRT1, SRY, GnRH1, FSHR*, and *ESR1*. FSH and LH were not significantly changed. Cortisol was not measured; T:C ratio could not be calculated.	Moderate
16	Evans et al. (18)	RCT crossover, triple-blind	16	Moderately stressed healthy adults	Single dose	L-theanine 200 mg, AlphaWave^®^, vs. placebo	Salivary cortisol decreased significantly more in the L-theanine arm at 1 h post-dose during mental arithmetic stress (p < 0.001). Frontal alpha oscillatory power increased. Testosterone was not measured; T:C ratio could not be calculated.	Low
17	White et al. (19)	RCT crossover, double-blind	34	Healthy adults, mixed sex	Single dose	L-theanine 200 mg-based drink vs. placebo	No cortisol difference was observed at 1 h post-dose; salivary cortisol response was significantly lower at 3 h post-dose (p = 0.047). Resting alpha activity increased. Testosterone was not measured; T:C ratio could not be calculated.	Some concerns
18	Moulin et al. (20)	RCT parallel, double-blind	30	Moderately stressed healthy adults	28 days	L-theanine 400 mg/day, AlphaWave^®^, vs. placebo	No significant between-group difference was observed in morning or evening resting salivary cortisol at Day 14 or Day 28. Perceived stress and Stroop reaction time improved. Testosterone was not measured; T:C ratio could not be calculated.	Low
19	Lim (21)	RCT parallel, randomized	18	Female college archery competitors	4 weeks	L-theanine vs. placebo, pre-competition	Salivary cortisol was significantly lower at 10 min pre-competition (p < 0.05). Salivary alpha-amylase was reduced at 20 and 10 min pre-competition, and cognitive anxiety decreased. Testosterone was not measured; T:C ratio could not be calculated.	Some concerns
20	Willems and Foster (57)	RCT crossover, double-blind	8 enrolled; 5 analyzed for HRV	Healthy young adult females	21 days	Encapsulated matcha, 3 g/day, providing 429 mg catechins, 90 mg caffeine, and 37 mg L-theanine, vs. placebo	Resting HR decreased by 13% (p = 0.01), RR intervals increased by 16% (p = 0.03), SDNN increased by 44% (p = 0.01), and pNN50 increased by 139% (p < 0.01), suggesting autonomic stress modulation via parasympathetic activation. Cortisol and testosterone were not measured; T:C ratio could not be calculated.	Low
21	Hintzpeter et al. (23)	Mechanistic *in vitro* evidence	Not applicable	Human liver microsomes and purified 11β-HSD1	*In vitro*	EGCG and green tea extract	EGCG inhibited 11β-HSD1 cortisone-to-cortisol reductase activity, and green tea extract also inhibited 11β-HSD1, identifying a prereceptor pathway for glucocorticoid modulation. Testosterone was not assessed; T:C ratio could not be calculated.	Not applicable, narrative appraisal
22	Szelényi et al. (24)	Mechanistic *in vitro* evidence	Not applicable	Liver microsomal cortisol-production system	*In vitro*	EGCG	Microsomal cortisol production was suppressed through a redox shift in the endoplasmic reticulum, a mechanism complementary to direct enzyme inhibition. Testosterone was not assessed; T:C ratio could not be calculated.	Not applicable, narrative appraisal

Extracted quantitative outcome data for the human studies, including baseline and follow-up values, measures of variability, effect estimates, units, and assessment time points, are reported in Supplementary Table S10. CE/TA, caffeine-plus-EGCG to L-theanine-plus-arginine molar ratio; DHEA, dehydroepiandrosterone; EGCG, epigallocatechin-3-gallate; FSH, follicle-stimulating hormone; HR, heart rate; LH, luteinizing hormone; pNN50, percentage of successive RR intervals differing by more than 50 ms; RCT, randomized controlled trial; RoB, risk of bias; ROS, reactive oxygen species; SDNN, standard deviation of normal-to-normal intervals; StAR, steroidogenic acute regulatory protein; T:C, testosterone-to-cortisol ratio. The 22 included studies comprise 15 human studies, five animal experiments, and two *in vitro* studies. The participant column reports the number enrolled or studied; where a given outcome was assessed in only a subset of those participants, the subset is stated. Human study components included 700 participants in total. Of these, 673 participants contributed to the endocrine-relevant human evidence base; Scholey et al. (56) was retained in the broader stress-related synthesis but did not contribute to this count. Animal and *in vitro* studies were interpreted as mechanistic evidence rather than direct clinical evidence. Hintzpeter et al. (23) and Szelényi et al. (24) are two independent publications by separate research groups and are therefore enumerated, appraised, and tabulated as two separate included studies. Their findings are complementary rather than duplicative, addressing direct inhibition of 11β-HSD1 reductase activity and suppression of microsomal cortisol production through an endoplasmic-reticulum redox shift, respectively. ACTH terminology is reproduced as reported in the source article. Because the source contains an unconventional expansion of this abbreviation, ACTH is presented without expansion in the narrative synthesis.

## Results

## Study selection

**Figure 2:**
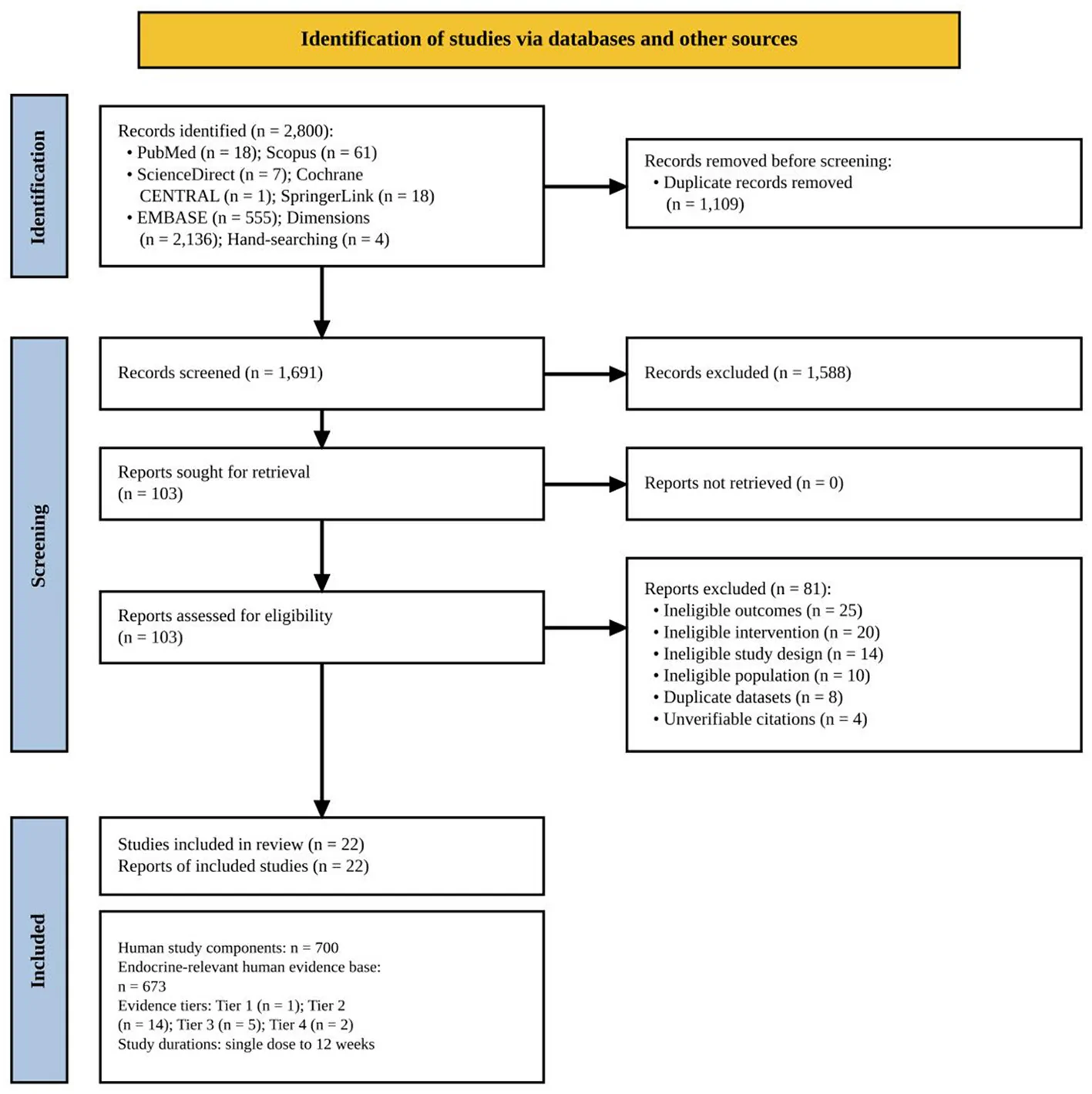
PRISMA 2020 flow diagram of study identification, screening, and inclusion. Records were identified through seven electronic sources and hand-searching of reference lists, merged before deduplication, and screened according to the prespecified eligibility criteria. Searches covered records from inception to 31 March 2026 and were restricted to English-language publications. The final synthesis included 22 studies. Adapted from the PRISMA 2020 statement (46).

A total of 2,800 records were identified (Table 2): 2,796 through database searches and four through targeted hand-searching of reference lists. After removal of 1,109 duplicates, 1,691 unique records underwent title and abstract screening, of which 1,588 were excluded. The remaining 103 reports were assessed in full text, and 81 were excluded because of ineligible outcomes, interventions, study designs, or populations; duplicate datasets; or unverifiable citations. Twenty-two studies met the prespecified eligibility criteria and were included in the synthesis (Figure 2).

## Characteristics of included studies, evidence tiers, and risk of bias

The included studies comprised randomized human intervention trials, non-randomized human studies, controlled animal experiments, and *in vitro* mechanistic models (Table 3). Applying the prespecified tier definitions, the 22 included studies comprised one Tier 1 direct clinical study, that is, a single human intervention administering whole matcha and measuring an endocrine endpoint (11); 14 Tier 2 supportive human studies; five Tier 3 animal experiments; and two Tier 4 *in vitro* studies. The scarcity of Tier 1 evidence is itself a principal finding of this review. Tier allocation for every study, together with the review aim or aims each study informs, is reported in Supplementary Table S9, and the extracted quantitative endocrine data, including baseline and follow-up values, effect estimates, measures of variability, units, and assessment time points, are reported in Supplementary Table S10. Human study components included 700 participants, of whom 673 contributed to the endocrine-relevant human evidence base. Scholey et al. (56) was retained in the broader stress-related synthesis but did not contribute to the endocrine-relevant count. Interventions ranged from acute single-dose assessments to regimens lasting up to 12 weeks and included whole matcha, green tea, decaffeinated green tea, green-tea catechins, isolated L-theanine, and related bioactive formulations. Most randomized trials were judged to be at low risk of bias for the assessed cortisol or stress-related outcomes, although some concerns remained regarding reporting, intervention composition, allocation procedures, or selective-reporting safeguards (Table 4; Supplementary Tables S2–S5). The uncontrolled pre-post study had a serious overall risk of bias, and the cross-sectional study had moderate concerns. Animal and *in vitro* studies were considered mechanistic support rather than direct clinical evidence.

**Table 4:** Risk of bias summary (Cochrane RoB 2 for RCTs; SYRCLE for animal studies).

References	Design	D1: Randomization	D2: Deviations from intended interventions	D3: Missing outcome data	D4: Outcome measurement	D5: Selection of reported result	Overall RoB
Kimura et al. (16)	RCT crossover	Low	Low	Low	Low	Some concerns	Some concerns
Yoto et al. (17)	RCT crossover	Low	Low	Low	Low	Low	Low
Scholey et al. (56)	RCT crossover	Low	Low	Low	Low	Low	Low
Suzuki et al. (63)	RCT crossover	Low	Low	Low	Low	Low	Low
Dini and Pourrazi (40)	RCT parallel	Some concerns	Some concerns	Some concerns	Low	Some concerns	Some concerns
Shigeta et al. (11)	RCT parallel	Low	Low	Low	Low	Low	Low
Sae-tan et al. (60)	Animal, mouse	See SYRCLE	See SYRCLE	See SYRCLE	See SYRCLE	See SYRCLE	Low
Chen et al. (45)	Animal, rat	See SYRCLE	See SYRCLE	See SYRCLE	See SYRCLE	See SYRCLE	Low
Almudhi and Gabr (35)	Pre-post intervention study, no comparator	Not applicable	Not applicable	Not applicable	Not applicable	Not applicable	Serious, ROBINS-I
Onuora et al. (64)	Pre-post interventional study, no placebo	Not applicable	Not applicable	Not applicable	Not applicable	Not applicable	High, ROBINS-I
Wan et al. (65)	Cross-sectional controlled study	Not applicable	Not applicable	Not applicable	Not applicable	Not applicable	Moderate, NOS
Unno et al. (13)	Human RCT and animal experiment	Low	Low	Low	Low	Low	Low
Monobe et al. (59)	Animal, mouse	See SYRCLE	See SYRCLE	See SYRCLE	See SYRCLE	See SYRCLE	Moderate
Mohamed et al. (58)	Animal, rabbit	See SYRCLE	See SYRCLE	See SYRCLE	See SYRCLE	See SYRCLE	Moderate
Soliman et al. (66)	Animal, rabbit	See SYRCLE	See SYRCLE	See SYRCLE	See SYRCLE	See SYRCLE	Moderate
Evans et al. (18)	RCT crossover, triple-blind	Low	Low	Low	Low	Low	Low
White et al. (19)	RCT crossover, double-blind	Low	Low	Low	Low	Some concerns	Some concerns
Moulin et al. (20)	RCT parallel, double-blind	Low	Low	Low	Low	Low	Low
Lim (21)	RCT parallel, randomized	Low	Low	Low	Low	Some concerns	Some concerns
Willems and Foster (57)	RCT crossover, double-blind	Low	Low	Low	Low	Low	Low
Hintzpeter et al. (23)	Mechanistic *in vitro* study	Not applicable	Not applicable	Not applicable	Not applicable	Not applicable	Not applicable, *in vitro*
Szelényi et al. (24)	Mechanistic *in vitro* study	Not applicable	Not applicable	Not applicable	Not applicable	Not applicable	Not applicable, *in vitro*

NOS, Newcastle-Ottawa Scale; RCT, randomized controlled trial; RoB, risk of bias; ROBINS-I, Risk of Bias in Non-randomized Studies of Interventions; SYRCLE, Systematic Review Center for Laboratory Animal Experimentation. D1–D5 refer to Cochrane RoB 2 domains and were applied only to randomized controlled trials. Animal studies were assessed using the SYRCLE risk-of-bias tool, with full domain-level ratings provided in Supplementary Table S2. The cross-sectional study by Wan et al. was assessed using an adapted Newcastle-Ottawa Scale, with full domain scores provided in Supplementary Table S3. The uncontrolled pre-post interventional study by Onuora et al. and the nonrandomized pre-post study by Almudhi and Gabr were both appraised using ROBINS-I rather than RoB 2, with full domain-level ratings provided in Supplementary Table S4a. The uncontrolled pre-post interventional study by Onuora et al. and the nonrandomized pre-post study by Almudhi and Gabr were both appraised using ROBINS-I rather than RoB 2, with full domain-level ratings provided in Supplementary Tables S4a and S4b, respectively. Cochrane RoB 2 domains were not applicable to animal, cross-sectional, uncontrolled pre-post, or mechanistic *in vitro* studies. Mechanistic *in vitro* studies were appraised narratively for methodological relevance and integrated into the GRADE indirectness assessment rather than assigned a formal clinical risk-of-bias rating. Unno et al. (13) reports both a human randomized trial and an animal experiment. The RoB 2 judgment shown applies to the human component. The animal component was appraised separately using SYRCLE and rated as having moderate concerns; full domain-level ratings are provided in Supplementary Table S2.

## Aim 1: effects on cortisol and hypothalamic-pituitary-adrenal axis-related outcomes

### Direct clinical evidence (Tier 1) and supportive human evidence (Tier 2)

Evidence concerning cortisol and HPA axis-related outcomes was context-dependent. In the 12-week resistance-training arm reported by Shigeta et al. (trial 2, *n* = 19 of the 36 participants across both trials; salivary sampling at a fixed time of day before and after the intervention), the change in salivary cortisol secretion rate was significantly lower in the matcha group than in the placebo group (*p* < 0.05) (11). In contrast, a one-week randomized clinical trial in young women with anxiety found that supplementation with 1,000 mg/day of green-tea leaf powder did not significantly alter serum cortisol relative to the control condition (*p* = 0.96) (40). In a six-week pre-post study, Almudhi and Gabr reported that six cups/day of decaffeinated green tea reduced cortisol, DHEA, ACTH, and corticosterone levels and increased the cortisol:DHEA ratio in adolescents, including those with moderate stuttering (35).

### Supportive human evidence, isolated L-theanine (Tier 2)

Studies of isolated L-theanine and L-theanine-containing formulations indicated variation according to stress context and the timing of biomarker collection. A 200 mg dose of L-theanine significantly reduced salivary cortisol 1 h after administration during an acute mental-arithmetic stressor (*p* < 0.001) (18). In a separate trial of an L-theanine-based nutrient drink, the salivary cortisol response to a cognitive stressor was reduced 3 h post-dose (*p* = 0.047) (19). Anticipatory salivary cortisol was reduced 10 min before a competitive archery event following L-theanine supplementation (*p* < 0.05) (21). By contrast, 28 days of L-theanine supplementation did not significantly alter resting salivary cortisol in adults with moderate stress (20). Kimura et al. (16) and Yoto et al. (17) reported attenuation of physiological stress responses following L-theanine intake during acute stress paradigms but did not provide direct cortisol measurements.

### Supportive human evidence, stress-related proxy endpoints (Tier 2)

Several studies reported stress-related proxy outcomes that were not treated as direct endocrine evidence. Matcha cookies providing 4.5 g/day of matcha with a low CE/TA ratio of 1.79 significantly reduced salivary α-amylase activity (*p* = 0.002) (13). Scholey et al. (56) reported stress-related cognitive and electroencephalographic effects following EGCG administration but did not measure salivary or serum cortisol. Willems and Foster reported changes in time-domain heart-rate-variability metrics following encapsulated matcha intake, in the 5 of 8 enrolled participants for whom heart-rate-variability data were analysable, but did not measure cortisol or testosterone (57).

### Animal and *in vitro* evidence (Tiers 3 and 4)

Preclinical and mechanistic studies provided supporting biological context. Animal studies reported lower plasma cortisol and insulin following matcha administration (58), reduced anxiety-like behaviors following continued matcha ingestion in stressed mice (59), and EGCG-mediated modulation of skeletal-muscle expression of genes related to fat oxidation in high-fat-fed mice (60). Two further animal reports were cited for mechanistic context but did not meet the endocrine eligibility criteria and were therefore not counted among the included studies: EGCG-related attenuation of stress-induced neural injury through PKCα and ERK1/2 signaling in rats (61), and L-theanine-related modulation of immune and neurotransmitter pathways in rats (62). Two independent *in vitro* studies identified EGCG-mediated inhibition of 11β-hydroxysteroid dehydrogenase type 1 as a candidate pathway for prereceptor cortisol modulation: Hintzpeter et al. (23) demonstrated inhibition of cortisone-to-cortisol reductase activity in human liver microsomes and with purified enzyme, and Szelényi et al. (24) demonstrated suppression of microsomal cortisol production through an endoplasmic-reticulum redox shift.

### Aim 2: effects on testosterone and hypothalamic-pituitary-gonadal axis-related outcomes

#### Supportive human and animal evidence (Tiers 2 and 3)

Evidence concerning testosterone preservation and hypothalamic-pituitary-gonadal (HPG) axis-related outcomes was derived mainly from non-matcha green-tea interventions, an uncontrolled human study, a cross-sectional observational study, and animal reproductive models. In a sprint-cycling protocol, plasma testosterone decreased significantly after exercise in the carbohydrate-only condition (*p* < 0.001) but was maintained when green-tea catechins were co-ingested, whereas cortisol increased after exercise in both conditions without a significant group effect (63). A 12-week uncontrolled pre-post study reported increased testosterone and reduced oxidative stress following green-tea intake in obese men (64). In a cross-sectional study of 280 middle-aged and older adult men, long-term green-tea consumption was associated with higher testosterone concentrations than non-consumption [574.66 (409.00–694.48) vs. 495.89 (362.26–608.95) ng/dL; *p* = 0.001] (65). Preclinical reproductive models indicated that matcha modulated testicular expression of reproductive genes, including *DMRT1, SRY*, and *GnRH1* (66). However, the increase in circulating testosterone in that model was most pronounced in the chia-treated group rather than the matcha-treated group, limiting direct inference regarding matcha-specific testosterone effects.

#### Aim 3: direct evidence for modulation of the testosterone-to-cortisol ratio

Although the available evidence addressed cortisol-related and testosterone-related outcomes separately, direct clinical evidence of integrated T:C ratio modulation remained insufficient. Studies assessing HPA axis-related outcomes generally did not measure concurrent testosterone, whereas studies evaluating HPG axis-related outcomes generally did not assess cortisol using compatible biological matrices and sampling frameworks (11, 13, 35, 40, 63–65). Direct clinical derivation of the T:C ratio was therefore not feasible. Owing to this methodological gap, heterogeneity in intervention formulations, and reliance on indirect evidence, GRADE certainty was rated as low for the individual cortisol and testosterone outcomes and very low for the integrated T:C ratio endpoint (Table 1).

#### Exploratory CE/TA stratification

Exploratory CE/TA stratification suggested that formulation characteristics may contribute to heterogeneity in stress-related outcomes (Supplementary Table S6). The matcha formulations evaluated by Unno et al. (13, 30) had CE/TA ratios of 1.79 and 1.80 and were associated with reduced salivary α-amylase activity and anxiety-related outcomes (30), whereas a comparator formulation with a CE/TA ratio of 10.64 did not show the same response (13). For Shigeta et al., only a partial upper-bound proxy of 2.83 could be derived because arginine was omitted from the denominator. This value was therefore not treated as an exact ratio or assigned to a categorical stratum (11). A complete CE/TA ratio could not be calculated for Willems and Foster because EGCG and arginine were not quantified separately (57). These analyses were exploratory and do not establish a clinically validated threshold.

#### Synthesis without meta-analysis

Meta-analysis and statistical assessment of publication bias were not undertaken because the included studies were not sufficiently comparable in terms of intervention composition, population, study design, biological matrix, sampling time, stress context, or outcome definition. The evidence was therefore synthesized without meta-analysis, distinguishing direct human endocrine evidence from stress-related proxy outcomes and mechanistic support.

## Discussion

This systematic review synthesized evidence from 22 studies, including randomized human intervention trials, non-randomized human studies, controlled animal experiments, and *in vitro* mechanistic models. The human study components involved 700 participants. The findings indicate that matcha, green tea, and selected tea-derived bioactives may influence cortisol-related and testosterone-related outcomes under specific conditions. However, the available evidence does not establish that matcha modulates the T:C ratio in humans. Most studies evaluated cortisol-related or testosterone-related outcomes separately, used different biological matrices and sampling frameworks, or examined isolated compounds rather than chemically characterized whole-matcha interventions (11, 13, 16–21, 35, 40, 58, 63–65).

The cortisol-related evidence was context-dependent. In a 12-week resistance-training trial, Shigeta et al. reported a lower change in salivary cortisol secretion rate in the matcha group than in the placebo group (11). Dini and Pourrazi found no significant change in serum cortisol after one week of green-tea leaf-powder supplementation in young women with anxiety (40). Almudhi and Gabr reported reductions in cortisol, DHEA, ACTH, and corticosterone and an increased cortisol:DHEA ratio following six weeks of decaffeinated green-tea consumption in adolescents (35). However, the latter study used a pre-post design and should be interpreted cautiously. A larger lifestyle trial that incorporated green tea within a broader dietary pattern likewise reported only modest effects on morning cortisol, although the contribution of green tea could not be isolated from the whole-diet intervention (67). Together, these studies do not support a uniform cortisol-lowering effect. Instead, they suggest that the observed response may depend on the intervention formulation, population, stress context, and timing of biomarker collection.

The L-theanine evidence further illustrates the importance of sampling context. Kimura et al. and Yoto et al. reported attenuation of physiological stress responses during acute stress paradigms, although neither study directly measured cortisol (16, 17). Evans et al. found that a single 200 mg dose of L-theanine reduced salivary cortisol 1 h after administration during an acute laboratory stress challenge (18). White et al. (19) reported a lower salivary cortisol response to a cognitive stressor 3 h post-dose, but not at the earlier assessment. Lim reported lower anticipatory salivary cortisol 10 min before a competitive archery event following L-theanine supplementation (21). By contrast, Moulin et al. (20) found no significant change in resting salivary cortisol after 28 days of supplementation in adults with moderate stress. These findings suggest that L-theanine-related effects may be more readily detectable during acute or anticipatory stress than under resting sampling conditions. They also demonstrate why a null result from a single resting time point should not be interpreted as evidence that the intervention has no effect on stress physiology.

Matcha-specific evidence remains limited but supports further investigation of the food matrix. Unno et al. (13) found that matcha cookies providing 4.5 g/day of matcha with a CE/TA ratio of 1.79 reduced salivary α-amylase activity in students. In a separate animal and clinical investigation, Unno et al. (30) linked lower CE/TA formulations with stress-related outcomes, including suppression of stress-associated adrenal hypertrophy in mice. Monobe et al. reported reduced anxiety-like behavior following continued matcha ingestion in stressed mice, although endocrine outcomes were not assessed (59). Mohamed et al. (58) reported lower plasma cortisol and insulin following matcha administration in rabbits. Soliman et al. further reported modulation of reproductive-gene expression in growing male rabbits, including DMRT1, SRY, and GnRH1 (66). Willems and Foster provided human evidence relevant to autonomic regulation by reporting changes in heart-rate-variability metrics following encapsulated matcha intake, but cortisol and testosterone were not measured (57). These studies support biological plausibility, but they do not provide direct clinical confirmation of modulation of the T:C ratio.

The testosterone-related evidence is more indirect and requires cautious interpretation. *In vitro* studies indicate that catechins can influence Leydig-cell steroidogenesis, but the direction of effect varies with the compound and experimental context (25, 26). Suzuki et al. reported maintenance of post-exercise testosterone when green-tea catechins were co-ingested with carbohydrate, whereas testosterone decreased in the carbohydrate-only condition (63). Because catechins were administered with carbohydrate, the observed preservation of testosterone cannot be attributed solely to catechin exposure. Onuora et al. (64) reported increased testosterone after 12 weeks of green-tea intake in obese men, although the increase reached significance only in the two hypogonadal subgroups, rising from 3.9 to 8.6 ng/mL in compensated hypogonadism (*p* = 0.020) and from 1.2 to 3.8 ng/mL in hypergonadotropic hypogonadism (*p* = 0.008), whereas the eugonadic obese subgroup showed no significant change (*p* = 0.429). The supplementation phase included 40 of the 100 screened participants, in four subgroups of 10. Wan et al. (65) found that long-term green-tea consumption was associated with higher testosterone concentrations in middle-aged and older men. The Wan study was cross-sectional and therefore cannot establish causality. Collectively, these studies provide supportive testosterone-related evidence but do not demonstrate that matcha directly increases testosterone.

Catechin-mediated modulation of testosterone metabolism may partly explain the apparently divergent findings. Jenkinson et al. demonstrated that green- and white-tea extracts and selected catechins suppress UGT2B17-mediated testosterone glucuronidation *in vitro* (27). As UGT2B17 contributes to androgen glucuronidation and urinary excretion, catechin exposure could theoretically influence testosterone clearance or bioavailability without increasing Leydig-cell production. This remains a mechanistic hypothesis rather than a clinically established effect. Nevertheless, it provides a plausible framework for interpreting why some human green-tea studies report preserved or higher testosterone concentrations while *in vitro* Leydig-cell studies report inhibitory effects under selected conditions (25–27, 63–65).

The reciprocal relationship between the HPA and hypothalamic-pituitary-gonadal (HPG) axes provides an additional integrative framework. Sustained glucocorticoid exposure can suppress reproductive signaling, including GnRH- and LH-related pathways, and impair testicular steroidogenesis (4, 68, 69). Reducing stress-related cortisol responses could therefore reduce inhibitory pressure on androgenic signaling. In parallel, catechin-related preservation of testosterone responses may support anabolic balance under selected conditions (63–65). These pathways provide a mechanistic rationale for studying the T:C ratio. However, the hypothesis remains unconfirmed because the included matcha-specific human trials did not measure testosterone and cortisol concurrently using compatible methods.

The exploratory CE/TA analysis provides a useful framework for interpreting heterogeneity among matcha formulations. CE/TA represents the molar ratio of caffeine and EGCG to L-theanine and arginine, and it was developed by Unno and colleagues, who also proposed the indicative thresholds discussed below (13, 30–32). The present review does not claim the index as a novel construct; its contribution is to apply an existing index prospectively as a stratification variable across a systematically assembled evidence base, and to show what that stratification does and does not resolve. The Unno studies suggest that the balance among these constituents may influence stress-related outcomes (13, 30). More recent compositional studies also show that infusion conditions and repeated brewing alter the ratio in the consumed beverage (31, 32). The threshold should not be treated as clinically validated. Earlier matcha studies linked favorable stress-related findings with ratios close to or below 2, whereas later infusion studies proposed that ratios below 3 may be compatible with stress-reducing effects (13, 30–32). These values reflect an evolving compositional model rather than an established clinical cutoff. Future matcha trials should report the complete chemical composition of the administered preparation, including caffeine, EGCG, L-theanine, and arginine, and should calculate CE/TA prospectively.

Caffeine is particularly relevant to the interpretation of CE/TA. Matcha contains caffeine, which can acutely stimulate cortisol secretion and modify the response to stress (70, 71). A favorable stress-related response to a low-CE/TA formulation may therefore reflect the balance between caffeine-related stimulation and the modulatory effects of L-theanine and arginine rather than the action of a single constituent (13–17, 30–32, 70, 71). This is one reason why results from isolated L-theanine, concentrated EGCG, brewed green tea, and whole matcha should not be treated as directly interchangeable.

The pharmacokinetic behavior of EGCG adds further complexity. EGCG has limited oral bioavailability, variable absorption, and rapid systemic clearance (28, 29). Whole-matcha consumption differs from steeped green tea because the leaf powder is consumed as a suspension, potentially altering intestinal exposure, matrix interactions, and microbiota-mediated biotransformation (10, 28, 29). Shigeta et al. reported matcha-associated changes in the abundance of several bacterial genera, including *Ruminococcus, Butyricimonas*, and *Oscillospira*, which were associated with resistance-training outcomes (11). These observations are preliminary and do not establish an endocrine mechanism. However, they support the inclusion of microbiome profiling and catechin-metabolite analysis in future trials.

Dose and delivery format are also important for safety interpretation. Findings from high-dose isolated EGCG preparations should not be extrapolated directly to dietary matcha. EFSA concluded that catechins from traditionally prepared green-tea infusions are generally considered safe at reported intake levels, whereas supplemental EGCG doses at or above 800 mg/day may increase serum transaminases (72). Future trials using concentrated EGCG supplements should monitor liver enzymes. This precaution should be distinguished clearly from the safety profile of typical dietary matcha intake.

The distinction between systemic cortisol secretion and local cortisol activation also warrants consideration. EGCG can inhibit 11β-hydroxysteroid dehydrogenase type 1 (11β-HSD1), which regenerates active cortisol from cortisone in metabolically active tissues (23, 24). Platero et al. (73) reported no significant change in total blood cortisol following EGCG plus coconut oil in patients with multiple sclerosis but discussed the possibility that altered cortisol binding could influence free cortisol activity. In obese and diabetic rat models, Atsarina et al. reported reductions in hepatic 11β-HSD1 expression and serum cortisol following combined metformin and EGCG treatment (74). Lee et al. (75) reported reduced depression- and anxiety-like behaviors following chronic catechin administration in a corticosterone-based rat model. These findings suggest that catechins may influence glucocorticoid biology through prereceptor metabolism and central stress-regulatory pathways rather than through simple suppression of adrenal secretion.

Chronobiology remains an important methodological limitation. Cortisol follows a pronounced diurnal rhythm, and the timing of sample collection can substantially affect interpretation (36, 38, 39). Morning, evening, resting, anticipatory-stress, and post-stress cortisol values represent different physiological states and should not be combined without careful consideration. Because the T:C ratio is a quotient of two independently perturbable hormones, these confounders, together with matrix choice, binding-protein status, assay platform, training and sleep history, and acute caffeine exposure, propagate directly into the index rather than canceling within it. Future trials should standardize sampling relative to waking time, define fixed sampling windows, and collect repeated measures when feasible. The biological matrix should also be prespecified. Serum and salivary measurements are not directly interchangeable, and testosterone and cortisol ratios can vary according to whether total or free hormone concentrations are used (36–39).

Several limitations should be acknowledged. First, only a limited number of whole-matcha studies directly measured endocrine outcomes, and paired testosterone and cortisol measurements were rare. Second, much of the evidence was indirect, relying on isolated L-theanine, concentrated EGCG, brewed green tea, catechin preparations, animal models, or *in vitro* experiments. Third, heterogeneity in populations, formulations, biological matrices, intervention durations, stress paradigms, and sampling times precluded meta-analysis and formal dose-response modeling. Fourth, CE/TA calculations were limited by incomplete reporting of intervention composition. For example, a partial upper-bound proxy could be calculated for Shigeta et al., but arginine was not quantified; a complete ratio could not be calculated for Willems and Foster because EGCG and arginine were not reported separately (11, 57). Fifth, female-specific evidence remains limited, and no included human trial evaluated the T:C ratio following standardized whole-matcha supplementation in women. Sixth, three features of the search may have introduced retrieval bias. Web of Science was not accessible during the search period, although the seven sources actually searched overlap substantially with its indexing scope and hand-searching yielded only four additional records, which suggests the search had approached saturation. Eligibility was restricted to English-language publications, which raises the possibility of language bias; this is a particular consideration for the present topic, because a substantial body of matcha research is published in Japanese. Trial registries and gray-literature sources were not searched, so unpublished or null-result trials may be under-represented, and this could bias the evidence base toward positive findings. Finally, corticosterone was not included as an explicit search term in all databases. Some peripheral animal studies may therefore have been missed, although this is unlikely to alter the principal human conclusions.

The GRADE ratings reflect these limitations. Certainty was rated as low for cortisol-related outcomes, low for testosterone-related outcomes, and very low for the integrated T:C endpoint. These ratings reflect indirectness, heterogeneity, imprecision, and the lack of paired hormone measurements. The findings should therefore be interpreted as hypothesis-generating rather than clinically confirmatory.

Future research should prioritize randomized controlled trials using chemically characterized whole-matcha preparations with verified caffeine, EGCG, L-theanine, arginine, and CE/TA content (7, 10, 13, 30–32). Trials should measure testosterone and cortisol prospectively within the same participants, prespecify the calculation method for the T:C ratio, standardize the biological matrix and sampling schedule, and use caffeine-matched and sensory-matched placebos (36–39, 70, 71). Where feasible, studies should include free and total cortisol, cortisol-binding proteins, 11β-HSD1-related markers, catechin-metabolite profiling, microbiome characterization, liver-enzyme monitoring for concentrated EGCG preparations, and sex-stratified analyses (10, 23, 24, 28, 29, 72–74).

## Conclusions

This systematic review provides a CE/TA-stratified synthesis of evidence evaluating matcha, green tea, and selected tea-derived bioactives in relation to cortisol, testosterone, and the T:C ratio. Across 22 studies, the evidence supports biological plausibility for endocrine stress regulation, particularly through context-dependent modulation of stress-related outcomes, catechin-related effects on prereceptor cortisol metabolism, and testosterone-related pathways. However, the evidence remains indirect for the integrated T:C endpoint because the included matcha-specific human interventions did not measure testosterone and cortisol concurrently using compatible sampling frameworks.

Current evidence does not establish matcha as a validated T:C-modulating intervention. Matcha should instead be considered a promising, formulation-dependent candidate for future endocrine-nutrition research. RCTs should use chemically characterized whole-matcha preparations, report caffeine, EGCG, L-theanine, arginine, and CE/TA content, use appropriate placebo controls, standardize cortisol sampling windows, and measure paired testosterone and cortisol outcomes prospectively. Until such trials are conducted, matcha should be regarded as a biologically plausible but clinically unconfirmed nutritional strategy for endocrine stress regulation.

## Data Availability

The original contributions presented in the study are included in the article/Supplementary material, further inquiries can be directed to the corresponding author.

## Supplementary material

Supplementary Table S1 Risk-of-bias and critical-appraisal approaches by study design.

Supplementary Table S2 SYRCLE appraisal of animal studies.

Supplementary Table S3 Newcastle-Ottawa Scale appraisal of the cross-sectional study.

Supplementary Table S4a,b ROBINS-I appraisal of the nonrandomized intervention studies.

Supplementary Table S5 Cochrane RoB 2 appraisal of randomized controlled trials.

Supplementary Table S6 CE/TA ratio calculations, classifications, and assumptions.

Supplementary Table S7 Documented search strategies, platforms, dates, filters and limits, and record counts by source.

Supplementary Table S8 Structured eligibility criteria by PICOS element.

Supplementary Table S9 Evidence-tier assignment and allocation of each included study to the three review aims.

Supplementary Table S10 Extracted quantitative endocrine outcome data, including baseline and follow-up values, effect estimates, measures of variability, units, and assessment time points.
