# Supplementary material for: A CE/TA-stratified systematic review and evidence map of matcha and green tea bioactives in endocrine stress regulation: testosterone, cortisol, and the T:C ratio
Source: Front Nutr. 2026 Sep 8;13:1925871. doi: 10.3389/fnut.2026.1925871 (PMC13595566; doi:10.3389/fnut.2026.1925871)
Supplement: Supplementary file 1 [file Supplementary_File_1.docx]

**Supplementary Materials**

**A CE/TA-Stratified Systematic Review and Evidence Map of Matcha and Green Tea Bioactives in Endocrine Stress Regulation: Testosterone, Cortisol, and the T:C Ratio**

INPLASY registration: INPLASY202640072

This supplementary file provides the structured eligibility criteria, the evidence-tier assignment for each of the 22 included studies, the study-design-specific risk-of-bias assessments, the CE/TA compositional stratification, the extracted quantitative outcome data, and the complete database search strategies used in the systematic review. Risk-of-bias tools were selected according to study design; therefore, animal, randomized, nonrandomized, observational, and mechanistic studies were appraised using different approaches as summarized below. Because the included studies were methodologically and clinically heterogeneous, evidence was synthesized narratively in accordance with the Synthesis Without Meta-analysis (SWiM) reporting guideline [54].

**Table S1. Risk-of-Bias and Critical Appraisal Tools Applied by Study Design**

| **Study type** | **Appraisal approach** | **Supplementary table** | **Interpretation** |
| --- | --- | --- | --- |
| Animal intervention studies | SYRCLE risk-of-bias tool | Table S2 | Used to assess internal validity in animal experiments across the complete 10-domain SYRCLE framework. |
| Cross-sectional observational study | Adapted Newcastle-Ottawa Scale | Table S3 | Used to assess selection, comparability, and outcome domains in the observational testosterone evidence. |
| Nonrandomized interventional study | ROBINS-I | Tables S4a and S4b | Used to assess risk of bias in the uncontrolled human intervention study. |
| Human randomized controlled trials | Cochrane Risk of Bias 2 tool | Table S5 | Used to assess outcome-specific risk of bias for primary cortisol or stress-related outcomes. |
| Studies with sufficient compositional data | CE/TA molar ratio stratification | Table S6 | Used as an exploratory framework to contextualize formulation-dependent heterogeneity. |
| In vitro or mechanistic studies | Narrative critical appraisal of basic methodological validity, including physiological relevance of dosing, integrated with the GRADE indirectness domain. | Not assigned to a formal clinical risk-of-bias table | Interpreted as mechanistic support rather than direct clinical evidence. |

*Abbreviations: CE/TA, caffeine-plus-EGCG to L-theanine-plus-arginine molar ratio; EGCG, epigallocatechin-3-gallate; GRADE, Grading of Recommendations, Assessment, Development and Evaluation; ROBINS-I, Risk Of Bias In Non-randomized Studies of Interventions; SYRCLE, Systematic Review Centre for Laboratory Animal Experimentation.*

**Table S2. SYRCLE Risk-of-Bias Assessment for Animal Studies**

| **Study** | **Sequence generation** | **Baseline characteristics** | **Allocation concealment** | **Random housing** | **Blinding of caregivers / investigators** | **Random outcome assessment** | **Blinding of outcome assessment** | **Incomplete outcome data** | **Selective outcome reporting** | **Other sources of bias** | **Reviewer-Derived Overall Interpretation** |
| --- | --- | --- | --- | --- | --- | --- | --- | --- | --- | --- | --- |
| Sae-tan et al., 2011 | Low | Low | Unclear | Low | Unclear | Unclear | Unclear | Low | Low | Low | Low concerns |
| Chen et al., 2010 | Low | Low | Low | Low | Unclear | Unclear | Unclear | Low | Low | Low | Low concerns |
| Unno et al., 2019 | Low | Low | Unclear | Low | Unclear | Unclear | Unclear | Low | Low | Low | Moderate concerns |
| Monobe et al., 2019 | Low | Low | Unclear | Unclear | Unclear | Unclear | Unclear | Low | Low | Low | Moderate concerns |
| Mohamed et al., 2025 | Low | Low | Unclear | Low | Unclear | Unclear | Unclear | Low | Low | Low | Moderate concerns |
| Soliman et al., 2025 | Low | Low | Unclear | Low | Unclear | Unclear | Unclear | Low | Low | Low | Moderate concerns |

*Tool: SYRCLE risk-of-bias tool adapted from Hooijmans et al. (2014).*

*Interpretation: "Unclear" indicates insufficient reporting to determine risk of bias. Reviewer-derived overall interpretations were based on the distribution and likely impact of domain-level concerns. The domains "blinding of caregivers/investigators" and "random outcome assessment" were included to reflect the complete 10-domain SYRCLE structure.*

**Table S3. Newcastle-Ottawa Scale Assessment for the Cross-Sectional Study**

| **Domain / item** | **Maximum score** | **Assigned score** | **Justification** |
| --- | --- | --- | --- |
| Selection |  |  |  |
| Representativeness of the sample | 1 | 1 | Community-based sample of middle-aged and older males; broadly representative of the target population of habitual green tea consumers. |
| Sample size | 1 | 1 | Sample size was adequate for the primary testosterone comparison. |
| Non-response | 1 | 0 | Detailed non-response characteristics were not fully described; the criterion for full credit was not clearly met, so no point was awarded. |
| Ascertainment of exposure | 2 | 1 | Green tea consumption was self-reported; one point was deducted because exposure was not validated using biomarkers or objective intake monitoring. |
| Comparability |  |  |  |
| Control for confounding | 2 | 2 | Analyses adjusted for relevant covariates, including age, BMI, lifestyle factors, and depression-related variables. |
| Outcome |  |  |  |
| Outcome assessment | 2 | 2 | Testosterone was measured using a serum immunoassay, providing an objective biological endpoint. |
| Statistical test | 1 | 1 | Appropriate statistical comparisons and covariate-adjusted analyses were reported. |
| Total | 10 | 8 | Overall: moderate risk of bias, primarily due to self-reported exposure without biomarker validation. |

*Tool: Adapted Newcastle-Ottawa Scale for cross-sectional studies. Overall judgment: Moderate risk of bias.*

**Table S4a. ROBINS-I Assessment for the Testosterone-Related Outcome in the Nonrandomized Interventional Study**

| **ROBINS-I domain** | **Judgment** | **Justification** |
| --- | --- | --- |
| D1: Bias due to confounding | Serious | The study lacked a concurrent control group. Pre-existing hormonal status, dietary change, lifestyle modification, regression to the mean, and other time-related effects could not be excluded. |
| D2: Bias in selection of participants | Moderate concerns | Participants were recruited in a clinical setting, and variation in baseline gonadal or metabolic status may have influenced endocrine outcomes. |
| D3: Bias in classification of interventions | Low | The intervention was clearly defined as green tea consumed twice daily for 12 weeks and appeared to be consistently applied. |
| D4: Bias due to deviations from intended interventions | Low | No major deviations or relevant co-interventions were identified; compliance was reported as adequate. |
| D5: Bias due to missing data | Low | Outcome data were reported for all participants. |
| D6: Bias in measurement of outcomes | Moderate concerns | Testosterone was measured objectively, but lack of blinding to time point and the pre-post design may have introduced measurement or expectation-related bias. |
| D7: Bias in selection of the reported result | Moderate concerns | The study was not prospectively registered, and selective emphasis on statistically significant findings could not be excluded. |
| Overall judgment | Serious | The absence of a concurrent control group was the dominant limitation. Findings were interpreted as supportive but non-confirmatory evidence for testosterone-related effects. |

*Tool: ROBINS-I adapted from Sterne et al. (2016). Abbreviation: ROBINS-I, Risk of Bias in Non-randomized Studies of Interventions.*

**Table S4b. ROBINS-I Assessment for the Endocrine (Cortisol-Related) Outcome in the Nonrandomized Pre-Post Intervention Study (Almudhi and Gabr, 2022)**

| **ROBINS-I domain** | **Judgment** | **Justification** |
| --- | --- | --- |
| D1: Bias due to confounding | Serious | No untreated or placebo comparator was included; the healthy group also received the intervention. Pre-post changes cannot be confidently separated from time-related effects, regression to the mean, maturation, behavioral changes, or other uncontrolled influences. Baseline BMI and waist-hip ratio also differed between groups. |
| D2: Bias in selection of participants | Moderate concerns | Recruitment and eligibility criteria were described in part, but the complete recruitment and exclusion flow was not reported. |
| D3: Bias in classification of interventions | Low | The intervention was clearly defined as six cups/day of decaffeinated green tea for 6 weeks. |
| D4: Bias due to deviations from intended interventions | Moderate concerns | Participants were instructed not to change their usual habits and to avoid caffeine-containing drinks or drugs that could affect the results, but adherence was not objectively quantified and the study was unblinded. |
| D5: Bias due to missing data | Low | Adrenal hormones were reported for all participants, with no clear evidence of substantial missing data, although attrition reporting was limited. |
| D6: Bias in measurement of outcomes | Low | Serum biomarkers were measured objectively using standardized assays, with blood collected at 8:30 AM before and after the intervention. |
| D7: Bias in selection of the reported result | Moderate concerns | No preregistered protocol or statistical-analysis plan was reported, so selective analysis or reporting cannot be fully excluded. |
| Overall judgment | Serious | The absence of an untreated or placebo comparator was the dominant limitation, driving the overall rating to serious risk of bias. Endocrine findings were interpreted as supportive but non-confirmatory. |

*Tool: ROBINS-I adapted from Sterne et al. (2016). Abbreviation: ROBINS-I, Risk of Bias in Non-randomized Studies of Interventions.*

**Table S5. Cochrane RoB 2 Assessment for Human Randomized Controlled Trials**

| **Study** | **Design** | **Randomization process** | **Deviations from intended interventions** | **Missing outcome data** | **Measurement of outcome** | **Selection of reported result** | **Overall judgment** |
| --- | --- | --- | --- | --- | --- | --- | --- |
| Evans et al., 2021 | Randomized crossover, triple-blind | Low | Low | Low | Low | Low | Low |
| White et al., 2016 | Randomized crossover, double-blind | Low | Low | Low | Low | Some concerns | Some concerns |
| Moulin et al., 2024 | Randomized parallel-group, double-blind | Low | Low | Low | Low | Low | Low |
| Lim, 2024 | Randomized parallel-group trial | Low | Low | Low | Low | Some concerns | Some concerns |
| Willems and Foster, 2024 | Randomized crossover, double-blind | Low | Low | Low | Low | Low | Low |
| Shigeta et al., 2023 | Randomized parallel-group | Low | Low | Low | Low | Low | Low |
| Unno et al., 2019 (human component) | Randomized controlled trial; human component of a combined human and animal study | Low | Low | Low | Low | Low | Low |
| Dini and Pourrazi, 2025 | Randomized parallel-group | Some concerns | Some concerns | Some concerns | Low | Some concerns | Some concerns |
| Kimura et al., 2007 | Randomized crossover | Low | Low | Low | Low | Some concerns | Some concerns |
| Scholey et al., 2012 | Randomized crossover, double-blind | Low | Low | Low | Low | Low | Low |
| Suzuki et al., 2015 | Randomized crossover | Low | Low | Low | Low | Low | Low |
| Yoto et al., 2012 | Randomized crossover | Low | Low | Low | Low | Low | Low |

*Tool: Cochrane Risk of Bias 2 tool.*

*Note: Cochrane RoB 2 assessments were conducted specifically for the primary cortisol or stress-related outcome reported in each respective trial, rather than the study as a whole. For randomized crossover trials, the RoB 2 crossover-trial variant was applied, including consideration of period and carryover effects.*

*Note on Kimura et al. (2007), Yoto et al. (2012), Scholey et al. (2012), and Suzuki et al. (2015): These earlier trials appear in main Table 4 with RoB 2 domain-level judgments derived from a narrative appraisal using RoB 2 domain criteria. They are now included in this table for completeness, with domain-level judgments consistent with those reported in main Table 4. This is a transparent methodological distinction and does not change the cortisol GRADE participant count (N = 214 from Dini and Pourrazi, Almudhi and Gabr, Shigeta, Evans, White, Moulin, and Lim), in which Kimura, Yoto, and Unno are not included because cortisol was not a primary measured endpoint in those trials. Note on Unno et al. (2019): this publication reports both a human randomized trial and an animal experiment. The human component is appraised here with RoB 2; the animal component is appraised separately with SYRCLE in Table S2, where it is rated as having moderate concerns. The two components are therefore rated independently and should not be combined into a single judgment.*

*Interpretation: White et al. and Lim were judged as having "some concerns" because of limited reporting related to allocation concealment, co-ingredients, or selective reporting safeguards. All other randomized trials in this table were judged as low risk of bias for the assessed outcome.*

**Table S6. CE/TA Molar-Ratio Stratification of Included Studies**

**CE/TA ratio formula:**$CE/TA =\frac{moles of caffeine + moles of EGCG}{moles of L-theanine + moles of arginine}$

| **Study / intervention** | **CE/TA ratio** | **CE/TA classification** | **Cortisol or stress-related outcome** | **Testosterone outcome** | **Interpretation** |
| --- | --- | --- | --- | --- | --- |
| Unno et al., 2019 [13], test-matcha | 1.79 | Below threshold, ≤2.0 | Reduced salivary α-amylase activity, p = 0.002 | Not measured | Stress-related improvement was observed with a low-ratio matcha formulation. |
| Unno et al., 2018 [30], matcha tea | 1.80 | Below threshold, ≤2.0 | Reduced salivary α-amylase activity, p = 0.03; reduced anxiety | Not measured | Stress-related improvement was observed with a low-ratio matcha formulation. |
| Shigeta et al., 2023 [11], matcha beverage | 2.83 calculated without arginine | Partially estimated upper-bound proxy; category not assigned | Reduced salivary cortisol secretion rate, p < 0.05 | Not measured | A complete CE/TA ratio could not be calculated because arginine was not quantitatively reported. The partial proxy should not be treated as an exact ratio. |
| Willems and Foster, 2024 [57], encapsulated matcha | Non-calculable | Category not assigned | Cortisol not measured; heart-rate-variability metrics improved | Not measured | Autonomic benefit was reported, but CE/TA classification was not possible because EGCG and arginine were not quantified separately. |
| Unno et al., 2019 [13], placebo-matcha | 10.64 | Above threshold, >2.0 | No significant salivary α-amylase effect | Not measured | The higher-ratio comparator did not produce the same stress-related effect observed with the low-ratio formulation. |
| Kimura et al., 2007 [16] | Not applicable | Isolated compound | Physiological stress responses attenuated | Not measured | Isolated L-theanine intervention; the CE/TA framework is not applicable. |
| Evans et al., 2021 [18] | Not applicable | Isolated compound | Cortisol reduced by 42.4%, p < 0.001 | Not measured | Acute cortisol reduction was observed, but the intervention is not directly classifiable using CE/TA. |
| White et al., 2016 [19] | Not applicable | Multi-ingredient intervention | Cortisol reduced at 3 h, p = 0.047; not significant at 1 h | Not measured | A time-dependent cortisol effect was observed; interpretation is limited by the multi-ingredient design. |

**Table S6. Continued**

| **Study / intervention** | **CE/TA ratio** | **CE/TA classification** | **Cortisol or stress-related outcome** | **Testosterone outcome** | **Interpretation** |
| --- | --- | --- | --- | --- | --- |
| Lim, 2024 [21] | Not applicable | Isolated compound | Reduced pre-competition cortisol, p < 0.05 | Not measured | Cortisol reduction was observed in an ecological stress setting. |
| Moulin et al., 2024 [20] | Not applicable | Isolated compound | No significant change in resting cortisol | Not measured | The null finding may reflect the absence of an acute stress challenge. |
| Almudhi and Gabr, 2022 [35] | Non-calculable | Decaffeinated green tea | Reduced cortisol, DHEA, and ACTH, p < 0.05 | Not measured | Findings may reflect catechin-related or decaffeinated green-tea effects; CE/TA could not be calculated. |
| Onuora et al., 2025 [64] | Non-calculable | Green tea | Not measured | Increased testosterone, p < 0.05 | Relevant human testosterone evidence; the nonrandomized design limits causal inference. |
| Suzuki et al., 2015 [63] | Non-calculable | Catechins plus carbohydrate | Cortisol not altered | Testosterone maintained relative to a decline in the control group | Exercise-related endocrine evidence; not matcha-specific. |
| Dini and Pourrazi, 2025 [40] | Non-calculable | Green-tea leaf powder, 1,000 mg/day | No significant change (p = 0.96) | Not measured | CE/TA non-calculable; cortisol primary outcome; one week; young anxious women. |
| Wan et al., 2025 [65] | Not applicable | Observational green-tea exposure | Not measured | Higher testosterone among habitual green-tea consumers | Cross-sectional evidence; causal interpretation is not appropriate. |

*Abbreviations: CE/TA, caffeine-plus-EGCG to L-theanine-plus-arginine molar ratio; DHEA, dehydroepiandrosterone; EGCG, epigallocatechin-3-gallate. ACTH terminology is reproduced as reported in the source article. Because the source contains an unconventional expansion of this abbreviation, ACTH is presented without expansion in the narrative synthesis.*

*Classification rules: CE/TA values were classified as exact when all four constituent values were reported directly or when the complete ratio was reported by the original study. Values were classified as fully estimated when a complete ratio could be derived from study-specific compositional data. Values were classified as partially estimated when one or more constituents were unavailable and as non-calculable when the reported information was insufficient. Standard compositional averages were not used to impute missing values. Only exact or fully estimated ratios were assigned to categorical CE/TA strata.Note on Shigeta et al. (2023): A partial upper-bound proxy of 2.83 was calculated from the reported caffeine, EGCG, and L-theanine values. Arginine was not quantitatively reported. Because the inclusion of arginine would increase the denominator, the complete ratio would be lower than or equal to 2.83. The proxy was therefore not used for categorical stratification. Note on Willems and Foster (2024): This intervention was evaluated separately using its study-specific formulation data. Total catechins, caffeine, and L-theanine were reported, but EGCG and arginine were not quantified separately. A complete CE/TA ratio could therefore not be calculated.*

*Interpretation: CE/TA stratification was exploratory and hypothesis-generating. It was used to contextualize formulation-dependent heterogeneity rather than to establish a validated clinical threshold.*

**Table S7. Full Database Search Strategies**

*Search strategies were reported in accordance with the PRISMA-S extension for literature-search reporting [47]. The full executed search strings are reproduced below for each source. Hand-searching of reference lists is reported as a separate row, and the retrieved-record counts reconcile with the PRISMA flow diagram (Figure 2).*

| **Source** | **Platform or interface** | **Date searched** | **Full executed search string** | **Filters and limits applied** | **Records (n)** |
| --- | --- | --- | --- | --- | --- |
| PubMed/MEDLINE | NCBI PubMed (pubmed.ncbi.nlm.nih.gov) | 18 January 2026 | (("Tea"[Mesh] OR "Camellia sinensis"[tiab] OR "green tea"[tiab] OR matcha[tiab]) AND ("epigallocatechin gallate"[Supplementary Concept] OR EGCG[tiab] OR epigallocatechin[tiab] OR theanine[tiab] OR catechin[tiab] OR "green tea extract"[tiab]) AND ("Testosterone"[Mesh] OR "Hydrocortisone"[Mesh] OR testosterone[tiab] OR cortisol[tiab] OR "testosterone-to-cortisol ratio"[tiab] OR "T:C ratio"[tiab] OR "HPA axis"[tiab] OR "HPG axis"[tiab] OR stress[tiab] OR anabolic[tiab] OR "hydroxysteroid dehydrogenase"[tiab])) | Language: English. No date limit, no publication-type filter, no species filter, no article-type filter. | 18 |
| Scopus | Elsevier Scopus (scopus.com) | 20 January 2026 | TITLE-ABS-KEY(("matcha" OR "EGCG" OR "L-theanine" OR "green tea" OR "Camellia sinensis") AND ("testosterone" OR "cortisol" OR "T:C ratio" OR "hydroxysteroid dehydrogenase" OR "adrenal" OR "stress hormones")) | Language: English. No date limit, no document-type limit, no subject-area limit, no source-type limit. | 61 |
| ScienceDirect | Elsevier ScienceDirect (sciencedirect.com) | 22 January 2026 | Search 1: "matcha" AND ("testosterone" OR "cortisol") in Title, abstract, keywords. Search 2: "green tea" AND "cortisol" AND "endocrine" in Title, abstract, keywords. Results of both searches were merged before deduplication. | Language: English. No year limit, no article-type limit, no subject-area limit. Boolean operator count kept within the platform limit of eight, which is why the strategy was run as two complementary searches. | 7 |
| Cochrane CENTRAL | Cochrane Library (cochranelibrary.com) | 22 January 2026 | ("green tea" OR "matcha") AND "cortisol", searched across All Text. | No date limit. Trials segment only (CENTRAL); Cochrane Reviews and protocols not retrieved. No language filter applied at the platform; non-English records excluded at screening. | 1 |

**Table S7. Continued**

| **Source** | **Platform or interface** | **Date searched** | **Full executed search string** | **Filters and limits applied** | **Records (n)** |
| --- | --- | --- | --- | --- | --- |
| SpringerLink | Springer Nature SpringerLink (link.springer.com) | 24 January 2026 | ("Camellia sinensis" OR "green tea" OR "matcha") AND ("testosterone" OR "cortisol" OR "HPA axis" OR "stress hormones") | Language: English. Content type: Article. No date limit, no discipline filter. | 18 |
| EMBASE | Elsevier Embase (embase.com) | 10 February 2026 | (matcha OR EGCG OR theanine OR "green tea") AND (testosterone OR cortisol OR "testosterone:cortisol" OR "adrenal hormones") | Language: English. No date limit, no publication-type limit, no EMTREE explosion restriction. Broad retrieval retained deliberately to maximize sensitivity. | 555 |
| Dimensions | Digital Science Dimensions (app.dimensions.ai) | 12 February 2026 | (matcha OR "green tea" OR "Camellia sinensis") AND (EGCG OR epigallocatechin OR theanine OR catechin) AND (testosterone OR cortisol OR "hydroxysteroid dehydrogenase"), searched in full data (title, abstract, and full text where indexed). | Language: English. No date limit, no research-category limit, no publication-type limit. Full-text indexing accounts for the large retrieval. | 2,136 |
| Hand-searching | Reference lists of included studies and of matcha-focused reviews | Ongoing to 31 March 2026 | Manual screening of the reference list of every included study and of relevant matcha-focused systematic and narrative reviews identified during screening. | Not applicable. Records assessed against the same eligibility criteria. | 4 |
| Web of Science Core Collection | Clarivate (not accessible) | Not applicable | Planned in the registered protocol but not executed. | Institutional access was unavailable throughout the search period. This is disclosed as a protocol deviation and as a limitation. | Not applicable |
| Total |  | Final search 31 March 2026 |  |  | 2,800 |

*Abbreviations: EGCG, epigallocatechin-3-gallate; HPA, hypothalamic-pituitary-adrenal; HPG, hypothalamic-pituitary-gonadal; T:C, testosterone-to-cortisol ratio.*

**Table S8. Structured Eligibility Criteria by PICOS Element**

| **PICOS element** | **Inclusion criterion** | **Exclusion criterion** | **Operational rule applied** |
| --- | --- | --- | --- |
| Population | Human participants of any age, sex, or health status. Animal models of any species. In vitro human or animal tissue, microsomal, or cell-culture systems. | None on demographic grounds. Studies in which no biological system was studied, such as purely analytical or compositional reports. | Compositional and analytical papers were used only as contextual citations and were excluded from the endocrine evidence count. |
| Intervention | Whole matcha; matcha-containing foods or beverages; standardized Camellia sinensis preparations; brewed green tea; decaffeinated green tea; green-tea extracts; green-tea polyphenols; isolated EGCG, L-theanine, or caffeine. | Black, oolong, white, or herbal teas without a green-tea or matcha arm. Multi-ingredient products in which no tea-derived constituent could be attributed. | Multi-ingredient formulations were eligible only where the tea-derived constituent was quantified; they were flagged as multi-ingredient in Table S6. |
| Comparator | Placebo, vehicle, water, no intervention, alternative beverage, untreated control group, or within-subject pre-intervention baseline. | None. Uncontrolled pre-post designs were eligible but downgraded in appraisal. | Absence of a concurrent comparator was recorded as serious confounding under ROBINS-I rather than as grounds for exclusion. |
| Outcomes | Primary: serum or salivary testosterone, cortisol, or the T:C ratio. Secondary and mechanistic: HPA-axis or HPG-axis markers, corticosterone, DHEA, 11-beta-HSD1 activity, steroidogenic gene or protein expression, salivary alpha-amylase, heart-rate variability, and validated stress or anxiety measures. | Studies reporting no endocrine, stress-physiological, or mechanistically endocrine-relevant outcome. | Studies reporting only stress-related proxy endpoints were retained as Tier 2 supportive evidence and were excluded from the endocrine-relevant participant total where neither cortisol nor testosterone was measured. |
| Study design | Randomized controlled trials including crossover designs; nonrandomized and uncontrolled pre-post intervention studies; cross-sectional and other observational studies; controlled animal experiments; mechanistic in vitro studies. | Narrative reviews, systematic reviews, editorials, commentaries, conference abstracts without extractable primary data, case reports, and protocols without results. | Reviews were screened for reference-list hand-searching but were not included as studies. |
| Report characteristics | Peer-reviewed publications reporting extractable primary data, published in English, from database inception to 31 March 2026. | Non-English publications; records without retrievable full text; duplicate reports of the same dataset; records whose citation could not be verified. | Where several reports described one dataset, the most complete report was retained and the others were logged as duplicate datasets. |

*Abbreviations: DHEA, dehydroepiandrosterone; EGCG, epigallocatechin-3-gallate; HPA, hypothalamic-pituitary-adrenal; HPG, hypothalamic-pituitary-gonadal; PICOS, population, intervention, comparator, outcomes, study design; T:C, testosterone-to-cortisol ratio.*

**Table S9. Evidence-Tier Assignment and Allocation of Each Included Study to the Review Aims**

| **Study** | **Design** | **Evidence tier** | **Basis for tier assignment** | **Review aim informed** | **Counted in the endocrine-relevant human base (n)** |
| --- | --- | --- | --- | --- | --- |
| Shigeta et al., 2023 [11] | Human RCT, parallel | Tier 1: direct clinical | Whole matcha beverage with a measured endocrine endpoint (salivary cortisol secretion rate). Cortisol was measured in trial 2 only (n = 19 of the 36 participants across both trials). | Aim 1 | Yes (36 enrolled; 19 with cortisol) |
| Unno et al., 2019 [13] | Human RCT plus animal experiment | Tier 2: supportive human | Matcha-containing food, but the human endpoint was salivary alpha-amylase; cortisol and testosterone were not measured. The animal component was appraised with SYRCLE. | Aims 1 and 3 | Yes (36) |
| Willems and Foster, 2024 [57] | Human RCT, crossover | Tier 2: supportive human | Encapsulated matcha, but only autonomic endpoints were measured; heart-rate variability was analysable in 5 of the 8 enrolled participants. | Aim 1 | Yes (8 enrolled; 5 analysed) |
| Kimura et al., 2007 [16] | Human RCT, crossover | Tier 2: supportive human | Isolated L-theanine; physiological stress endpoints without direct cortisol assay. | Aim 1 | Yes (12) |
| Yoto et al., 2012 [17] | Human RCT, crossover | Tier 2: supportive human | Isolated L-theanine; blood-pressure and stress endpoints. | Aim 1 | Yes (14) |
| Scholey et al., 2012 [56] | Human RCT, crossover | Tier 2: supportive human | Isolated EGCG; no cortisol or testosterone was measured. The reported outcomes were cognitive, electroencephalographic, mood, and cardiovascular. | Aim 1 | No (27 excluded from the endocrine-relevant base) |
| Evans et al., 2021 [18] | Human RCT, crossover | Tier 2: supportive human | Isolated L-theanine with salivary cortisol under acute stress. | Aim 1 | Yes (16) |
| White et al., 2016 [19] | Human RCT, crossover | Tier 2: supportive human | L-theanine-based multi-ingredient drink with salivary cortisol. | Aim 1 | Yes (34) |
| Moulin et al., 2024 [20] | Human RCT, parallel | Tier 2: supportive human | Isolated L-theanine with resting salivary cortisol. | Aim 1 | Yes (30) |
| Lim, 2024 [21] | Human RCT, parallel | Tier 2: supportive human | Isolated L-theanine with anticipatory salivary cortisol. | Aim 1 | Yes (18) |
| Dini and Pourrazi, 2025 [40] | Human RCT, parallel | Tier 2: supportive human | Green-tea leaf powder with serum cortisol. | Aim 1 | Yes (20) |
| Almudhi and Gabr, 2022 [35] | Human uncontrolled pre-post | Tier 2: supportive human | Decaffeinated green tea with serum adrenal hormones. | Aim 1 | Yes (60) |
| Suzuki et al., 2015 [63] | Human RCT, crossover | Tier 2: supportive human | Green-tea catechins co-ingested with carbohydrate; paired testosterone and cortisol under exercise, but not a matcha preparation. | Aims 2 and 3 | Yes (9) |

**Table S9. Continued**

| **Study** | **Design** | **Evidence tier** | **Basis for tier assignment** | **Review aim informed** | **Counted in the endocrine-relevant human base (n)** |
| --- | --- | --- | --- | --- | --- |
| Onuora et al., 2025 [64] | Human uncontrolled pre-post | Tier 2: supportive human | Green tea with serum testosterone; no cortisol. 100 men were screened cross-sectionally and 40 received the 12-week supplementation in four subgroups of 10. | Aim 2 | Yes (100 screened; 40 supplemented) |
| Wan et al., 2025 [65] | Cross-sectional observational | Tier 2: supportive human | Habitual green-tea exposure with serum testosterone; no cortisol. | Aim 2 | Yes (280) |
| Sae-tan et al., 2011 [60] | Animal, mouse | Tier 3: animal | Dietary EGCG; metabolic and gene-expression endpoints. | Aim 1 (mechanistic) | Not applicable |
| Chen et al., 2010 [45] | Animal, rat | Tier 3: animal | Green-tea polyphenols and EGCG; stress-induced plasma cortisol. | Aim 1 (mechanistic) | Not applicable |
| Monobe et al., 2019 [59] | Animal, mouse | Tier 3: animal | Matcha and low-caffeine matcha; anxiety-like behavior after social stress. | Aim 1 (mechanistic) | Not applicable |
| Mohamed et al., 2025 [58] | Animal, rabbit | Tier 3: animal | Matcha in drinking water; plasma cortisol and insulin. | Aim 1 (mechanistic) | Not applicable |
| Soliman et al., 2025 [66] | Animal, rabbit | Tier 3: animal | Matcha in drinking water; testicular reproductive gene expression. | Aim 2 (mechanistic) | Not applicable |
| Hintzpeter et al., 2014 [23] | In vitro, microsomal and purified enzyme | Tier 4: in vitro | EGCG and green-tea extract; inhibition of 11-beta-HSD1 reductase activity. | Aim 1 (mechanistic) | Not applicable |
| Szelényi et al., 2013 [24] | In vitro, hepatic microsomes | Tier 4: in vitro | EGCG; suppression of microsomal cortisol production via an endoplasmic-reticulum redox shift. | Aim 1 (mechanistic) | Not applicable |

*Tier definitions. Tier 1, direct clinical evidence: human interventional studies administering whole matcha or a matcha-containing preparation in which testosterone, cortisol, or the T:C ratio was a measured endpoint. Tier 2, supportive human evidence: all other human studies, whether because the preparation was a non-matcha green-tea product or an isolated bioactive, or because only stress-related proxy endpoints were measured. Tier 3, animal evidence: controlled in vivo experiments. Tier 4, in vitro evidence: cell-free, microsomal, or cell-culture systems. Only Tiers 1 and 2 contributed to the human participant totals and to the GRADE assessment of human outcomes. Aims: Aim 1, cortisol and HPA-axis-related outcomes; Aim 2, testosterone and HPG-axis-related outcomes; Aim 3, direct evidence for modulation of the T:C ratio. Human study components totalled 700 participants, of whom 673 formed the endocrine-relevant base; Scholey et al. did not contribute to this base.*

**Table S10. Extracted Quantitative Endocrine and Stress-Related Outcome Data for the Human Studies**

| **Study** | **Outcome** | **Matrix** | **Assay** | **Assessment time points** | **Baseline or comparator value (mean plus or minus SD unless stated)** | **Follow-up value or between-group effect** | **p value** | **Confidence interval or effect size** | **Units** |
| --- | --- | --- | --- | --- | --- | --- | --- | --- | --- |
| Shigeta et al., 2023 [11] | Salivary cortisol secretion rate | Saliva | ELISA (Salimetrics) | Trial 2 only (n = 19); same time of day, before and after the 12-week intervention | Reported graphically (Fig. 4b) as mean plus or minus SD; numeric values not tabulated | Change in salivary cortisol secretion lower in the matcha group than in the placebo group | p < 0.05 | Not reported | Not stated |
| Dini and Pourrazi, 2025 [40] | Serum cortisol | Serum | Architect cortisol kit E-Abbott 8D15 (Abbott, Germany), automated analyser | Pre-intervention and after 1 week | Control 8.5 plus or minus 3.09; supplement 9.27 plus or minus 2.77 | Control 8.4 plus or minus 2.7 (within-group p = 0.74); supplement 9.14 plus or minus 2.78 (within-group p = 0.82); between-group mean difference 0.03 plus or minus 0.06 | p = 0.96 | Not reported | ug/dL |
| Almudhi and Gabr, 2022 [35] | Serum cortisol, DHEA, acetylcholine (abbreviated ACTH in the source), corticosterone, and the cortisol:DHEA ratio | Serum | RIA-ELISA (cortisol); SIA-ELISA (acetylcholine) | Before and after 6 weeks; control n = 30, moderate stuttering n = 30 | Reported graphically (Fig. 3); numeric values not tabulated | Decrease in cortisol, DHEA, acetylcholine, and corticosterone, with an increase in the cortisol:DHEA ratio in both groups | p = 0.01 (control); p = 0.001 (moderate stuttering) | Not reported | Not stated |
| Evans et al., 2021 [18] | Salivary cortisol | Saliva | Salimetrics high-sensitivity salivary cortisol ELISA | t = -1 h and t = +1 h around a single 200 mg dose and a mental arithmetic stressor; crossover with 7-day washout | L-theanine 5.95 plus or minus 3.87 (n = 15); placebo 5.46 plus or minus 2.86 (n = 16); between-group p = 0.951 | At +1 h, L-theanine 3.59 plus or minus 4.73 and placebo 3.36 plus or minus 2.00. Change from -1 h to +1 h: -2.35 plus or minus 2.23 versus -2.10 plus or minus 1.94; mean within-group change -42.4 percent versus -32.6 percent | p = 0.003 for the between-group change | Median (min to max) reported; no CI | mmol/L as reported |
| White et al., 2016 [19] | Salivary cortisol response to a multitasking stressor | Saliva | Salimetrics high-sensitivity salivary cortisol EIA (ELISA) | Pre- and post-stressor at 1 h and 3 h post-dose; crossover | Within-subject crossover; non-parametric change scores analysed | At 1 h no difference (z = 1.28; median change placebo -0.19, active -0.02). At 3 h lower with active treatment (z = -1.98; median change placebo 0.44, active -0.09) | p > 0.05 at 1 h; p = 0.047 at 3 h | Medians reported; no CI | Not stated |
| Moulin et al., 2024 [20] | Morning and evening salivary cortisol | Saliva | Not specified in the source | Baseline, day 14, and day 28; 400 mg/day; n = 15 per group | Placebo 17.05 plus or minus 7.36 versus L-theanine 12.64 plus or minus 9.58; baseline imbalance p = 0.028 | L-theanine -1.75 plus or minus 9.80 (-13.84 percent) at day 14 and -0.98 plus or minus 12.15 (-7.75 percent) at day 28; placebo -5.09 plus or minus 10.10 (-29.85 percent) and -5.02 plus or minus 10.02 (-29.44 percent) | All p > 0.05; no between-group difference | Not reported | nmol/L |

***Table S10. Continued***

| Study | Outcome | Matrix | Assay | Assessment time points | Baseline or comparator value (mean plus or minus SD unless stated) | Follow-up value or between-group effect | p value | Confidence interval or effect size | Units |
| --- | --- | --- | --- | --- | --- | --- | --- | --- | --- |
| Lim, 2024 [21] | Salivary cortisol | Saliva | Competitive enzyme immunoassay (ELISA), VersaMax microplate reader, Molecular Devices | 30, 20, and 10 min before competition and 20 min after competition | At pre-30: placebo 4.5 plus or minus 0.8; theanine 4.8 plus or minus 0.7 | Placebo 6.2 plus or minus 1.8, 14.6 plus or minus 3.8, and 18.9 plus or minus 4.8 versus theanine 7.6 plus or minus 1.2, 10.7 plus or minus 2.6, and 13.5 plus or minus 3.4 at pre-20, pre-10, and post-20 | Group F = 4.72 (p < 0.05); time F = 8.12 (p < 0.001); group by time F = 12.96 (p < 0.001) | Not reported | u/mL as reported |
| Suzuki et al., 2015 [63] | Plasma testosterone and cortisol | Plasma | Automated analyser (Hitachi 7020) with commercial kits (Randox kits) | Pre-exercise and 10 min post-exercise; crossover with trials 7 days apart at a fixed time of day | Reported graphically (Fig. 3); numeric values not tabulated | Testosterone was maintained when green tea was co-ingested with carbohydrate but decreased in the carbohydrate-only condition; cortisol rose comparably in both conditions | p < 0.001 for the post-exercise testosterone decrease in the carbohydrate-only condition | Not reported | Not stated |
| Onuora et al., 2025 [64] | Serum testosterone (also LH, FSH, prolactin, oestradiol) | Serum | ELISA | Baseline, 6 weeks, and 12 weeks; supplemented subgroups of n = 10 each | Eugonadic obese 5.5 plus or minus 1.8; compensated hypogonadism 3.9 plus or minus 1.0; hypergonadotropic hypogonadism 1.2 plus or minus 0.68 | At 12 weeks: 6.2 plus or minus 1.5; 8.6 plus or minus 0.48; and 3.8 plus or minus 0.29 respectively. The increase was significant only in the two hypogonadal subgroups | p = 0.429; p = 0.020; p = 0.008 respectively | Not reported | ng/mL |
| Wan et al., 2025 [65] | Serum testosterone | Serum | Not specified in the source | Single cross-sectional measurement | Non-consumers 495.89 (362.26 to 608.95) | Tea consumers 574.66 (409.00 to 694.48); Z = -3.390 | p = 0.001 | Interquartile range reported in place of a CI | ng/dL |
| Unno et al., 2019 [13] | Salivary alpha-amylase (morning and evening) | Saliva | Enzymatic assay | Before and across a 15-day pharmacy placement | Reported graphically (Fig. 2); numeric values not tabulated | Morning salivary alpha-amylase lower with test matcha (CE/TA 1.79) than with the comparator matcha (CE/TA 10.64) during placement; a non-significant trend at university | p = 0.002 during placement; p = 0.078 at university | Not reported | Not stated |
| Willems and Foster, 2024 [57] | Resting heart rate and time-domain heart-rate variability | Not applicable | Polar V800 with Kubios HRV analysis | Supine rest after 3 weeks of 3 g/day; heart-rate variability analysed in 5 of the 8 participants | Placebo: heart rate 66 plus or minus 5; RR interval 925 plus or minus 86; SDNN 99 plus or minus 47; pNN50 29 plus or minus 19 | Matcha: heart rate 58 plus or minus 6; RR interval 1074 plus or minus 145; SDNN 137 plus or minus 53; pNN50 52 plus or minus 17 | p = 0.01; p = 0.03; p = 0.01; p < 0.01 | 95 percent CI reported for every metric; Cohen d = -1.45, 1.25, 0.76, and 1.28 | bpm; ms; ms; percent |

*All values were extracted directly from the primary publications and verified against the source text or tables. Entries recorded as not reported, or as not stated numerically, indicate that the value was not available in the source; entries recorded as reported graphically indicate that the outcome was presented only in a figure. The frequency of such entries, and in particular the near-absence of reported confidence intervals across the human trials, is itself a finding of this review and underpins the recommendation that future trials report complete descriptive statistics with effect sizes and precision estimates. Willems and Foster is the only included trial reporting a confidence interval for every outcome. Kimura et al. and Yoto et al. are not tabulated because neither measured cortisol or testosterone, and Scholey et al. is not tabulated for the same reason. Animal and in vitro studies are not tabulated here because their endpoints are not commensurable with the human outcome measures; their findings are summarized in main Table 3. Note on Almudhi and Gabr: the abbreviation ACTH is used in that source to denote acetylcholine, not adrenocorticotropic hormone, and is reproduced here exactly as defined by the authors. Abbreviations: CE/TA, caffeine-plus-EGCG to L-theanine-plus-arginine molar ratio; CI, confidence interval; DHEA, dehydroepiandrosterone; ELISA, enzyme-linked immunosorbent assay; FSH, follicle-stimulating hormone; LH, luteinising hormone; pNN50, percentage of successive RR intervals differing by more than 50 ms; RIA, radioimmunoassay; SD, standard deviation; SDNN, standard deviation of normal-to-normal intervals; SIA, sandwich immunoassay.*
